# Sweeping Surface Trap States under the Carpet: Cd^2+^ Inclusion in InAs Quantum Dots

**DOI:** 10.1021/acsnano.6c15005

**Published:** 2026-10-30

**Authors:** Habibul Arfin, Jordi Llusar, Carmelita Rodà, Satyaprakash Panda, Yurii P. Ivanov, Giorgio Divitini, Mirko Prato, Andrea Toma, Andrea Fratelli, Matteo L. Zaffalon, Sergio Brovelli, Ivan Infante, Luca De Trizio, Liberato Manna

**Affiliations:** †Nanochemistry, ‡Electron Spectroscopy and Nanoscopy, §Materials Characterization, ∥Clean Room Facility and ⊥Chemistry Facility, 121451 Istituto Italiano di Tecnologia, Via Morego 30, 16163 Genova, Italy; # Dipartimento di Scienza dei Materiali, 9305 Università degli Studi di Milano-Bicocca, Via R. Cozzi 55, 20125 Milano, Italy; ∇ BCMaterials, Basque Center for Materials, Applications, and Nanostructures, UPV/EHU Science Park, Leioa 48940, Spain

**Keywords:** III−V semiconductors, quantum dots, alloyed nanocrystals, short-wave infrared, InAs, bandgap tunability

## Abstract

We have investigated the effect of CdCl_2_ as an additive in the colloidal synthesis of amino-As-based InAs quantum dots (QDs). Our results demonstrate that this metal halide promotes the incorporation of Cd into the InAs QDs, leading to the formation of In­(Cd)As alloyed QDs featuring a narrower bandgap and enhanced photoluminescence (PL) compared to their undoped InAs counterparts. By varying the relative amount of CdCl_2_, it was possible to synthesize, in a one-pot process, QDs with PL tunable from ∼1000 to ∼1430 nm with a quantum yield as high as 17% at ∼1430 nm, without requiring any postsynthesis shell growth. These In­(Cd)As QDs feature longer exciton lifetimes compared to InAs QDs, due to suppressed ultrafast nonradiative trap pathways, and a fast biexciton lifetime that follows the expected linear scaling with nanocrystal volume. Density functional theory (DFT) calculations revealed that (i) In­(Cd)As alloys, which are thermodynamically unfavorable in the bulk phase, are stabilized by finite-size effects; (ii) the bandgap narrowing induced by Cd incorporation arises primarily from a pronounced upward shift of the valence band maximum (VBM); and (iii) this upward shift of the VBM also buries the surface trap states below the valence band edge, thereby accounting for the observed high PL efficiency in In­(Cd)As QDs. Overall, this work provides mechanistic insights into defect passivation in InAs QDs and establishes metal-halide-mediated growth as an effective strategy for infrared emitting InAs-based QDs.

## Graphical abstract

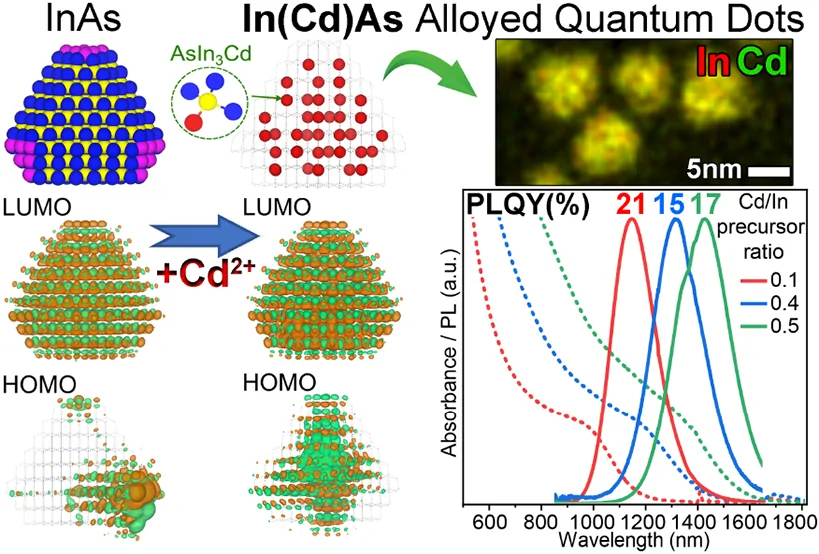

Colloidal semiconductor quantum dots (QDs) absorbing/emitting in the infrared (IR) spectral region are promising solution-processable materials for future consumer market IR products. IR applications of interest include light-emitting diodes and photodetectors for night and fog vision, food and object inspection systems, light detection and ranging, telecommunications, and biological imaging.^1–7^ Among IR QD materials, III–V semiconductors, in particular InAs QDs, are attracting increasing attention due to the tunability of their optical bandgap from the near IR up to the short-wave IR range by modulation of quantum confinement.^8–14^

To date, the most established route to synthesize colloidal InAs QDs is based on tris­(trimethylsilyl)­arsine (TMS-As).^15,16^ Given the pyrophoric nature, high cost, and limited market availability of such an As precursor, alternative synthetic approaches to InAs QDs have been explored in recent years. Among these, tris­(dimethylamino)­arsine (amino-As) has emerged as one of the most promising options due to its low cost and commercial availability.^9,17,18^ Indeed, in recent years, several groups have reported the successful synthesis of amino-As-based InAs QDs featuring an absorption edge tunable up to 2000 nm and emission up to 1500 nm.^19,20^ Despite significant progress, the synthesis of InAs QDs with efficient emission beyond 1000 nm remains a major challenge. This goal should ideally be pursued by first developing a simple synthesis route to large InAs QDs, preferably via a one-pot approach, followed by passivation of nonradiative surface traps to achieve high photoluminescence (PL) efficiency. In this regard, simple one-pot procedures reported to date have yielded amino-As-based InAs QDs that are either nonemissive or weakly emissive. For instance, Leemans et al. coupled amino-As with tris­(dimethylamino)­phosphine, the latter acting both as a precursor and a reducing agent, to prepare In­(As,P) QDs with a tunable absorption peak up to 1600 nm, but the PL efficiency was not reported.^21,22^ Kim et al. developed an amino-As-based synthesis procedure for InAs QDs that employs both diisobutylaluminum hydride and lithium bis­(trimethylsilyl)­amide and obtained QDs with absorption tunable up to 1700 nm; however, no PL was reported.^19^ Ginterseder et al. recently uncovered a one-pot synthesis route to large InAs QDs by employing In­(I)Cl as a key precursor, which acts as both the indium source and the reducing agent. However, the resulting InAs QDs featured only a weak PL beyond 1000 nm upon shelling with CdSe/CdS.^20^

To date, the only reports on amino-As-based InAs QDs with emission beyond 1000 nm rely either on a relatively complex seeded-growth approach,^18^ or a one-pot procedure based on Grignard reagents.^23^ In both cases, surface trap passivation was performed via ZnSe shelling, yielding InAs@ZnSe core@shell QDs. The former approach led to QDs with a PL quantum yield (QY) of ∼20% at a maximum wavelength of 1430 nm,^18^ whereas the latter achieved a PLQY of ∼6% at ∼1500 nm.^23^ Interestingly, in both works efficient PL could be obtained only when ZnCl_2_ was used as a synthesis additive.^12,17,23,24^ Indeed, as demonstrated by Zhu et al., such an additive results not only in an improved control over the size distribution of the InAs QDs, but also in the incorporation of Zn into the surface of the QDs.^24^ This in turn reduces surface-localized trap states with energies above the valence band maximum and originating from the dangling bonds of undercoordinated surface As ions present on the (−1–1–1) facets.^25–28^ These findings indicate that metal halide additives could potentially be exploited to enhance the optical properties of amino-As-based InAs QDs. However, the effect of metal halides other than ZnCl_2_ in InAs QD synthesis has remained largely unexplored.

This gap leads to the current study, in which we have investigated the effects of CdCl_2_ as an additive in the amino-As-based synthesis of InAs QDs. The choice of Cd was motivated by previous studies that established Cd as a dopant capable of inducing a transition from n-type to p-type behavior in InAs QDs when introduced in a postsynthesis treatment.^29,30^ Yet, in those studies, the influence of Cd on the synthesis process and, potentially, on the optical properties of InAs QDs was not investigated. Our results in this work indicate that the use of CdCl_2_ as a synthesis additive leads to the partial incorporation of Cd in the resulting InAs QDs, forming alloyed In–Cd–As systems (In­(Cd)As QDs), as suggested by structural and elemental analyses. Also, the optical absorption of the resulting QDs could be tuned from ∼950 to 1400 nm in a one-pot approach by modulation of the relative CdCl_2_ amount. Compared to InAs QDs of the same size, the In­(Cd)As QDs feature: (i) a red-shifted absorption, (ii) PLQY values as high as 17% at 1426 nm with no need for a subsequent shelling step. Time-resolved PL and transient absorption spectroscopy (TAS) revealed that the exciton lifetime progressively increased upon Cd incorporation, with In­(Cd)­As QDs emitting at 1426 nm showing a PL lifetime of 60 ns, comparable to that of state-of-the-art InAs@ZnSe QDs, suggesting a more efficient suppression of ultrafast nonradiative trap-assisted recombination pathways in these systems. Additionally, the biexciton lifetime was found to be short (i.e., 50 ps) and correlated to the QD volume, in line with the expected linear scaling found in confined systems.^17^ These experimental results were corroborated by density functional theory (DFT) calculations, which indicated that Cd incorporation in the InAs QD lattice leads to (i) an upward energetic shift of both InAs conduction and valence bands, with the latter experiencing a larger shift, therefore causing the observed narrowing of the optical bandgap; (ii) pushing of molecular orbitals localized on surface undercoordinated As ions below the delocalized band-edge orbitals, thereby submerging surface trap states, which rationalizes the increased PLQY compared to pure InAs QDs.

Our work demonstrates that the use of metal halide additives in the synthesis of amino-As-based InAs QDs can be an effective strategy to modulate their optical properties and may serve as a key approach for further implementations in these systems.

## Results and Discussion

The synthesis of In­(Cd)As QDs and InAs QDs (with no CdCl_2_ addition) was performed following the procedure reported by Panda et al. for InAs QDs with minor modifications (see Figures S1 and S2 and Tables S1 and S2 of the Supporting Information, SI).^18^ In detail, InCl_3_ was dissolved in oleylamine and octadecene together with a desired amount of CdCl_2_. Different Cd/In precursor ratios, ranging from 0 to 0.5, were explored by fixing the InCl_3_ amount and by systematically adjusting that of CdCl_2_. The reaction mixture was heated to 240 °C, followed by the injection of amino-As and then the trioctylamine-AlH_3_ complex as the reducing agent. After this, the reaction temperature was set to 280 °C, and the growth of the QDs was allowed to proceed for 5 min.

Figure 1a,b shows the absorption and PL spectra of the QDs synthesized under such reaction conditions. When no Cd was employed in the synthesis of InAs QDs, their first excitonic absorption peak was observed at ∼950 nm (Figure 1a). Upon incremental addition of CdCl_2_, the absorption of In­(Cd)­As QDs systematically red-shifted (from ∼950 to ∼1400 nm) and broadened compared to the InAs case (Figure 1a and Table 1). The In­(Cd)As samples exhibited a bright PL that was tunable from 1100 to ∼1430 nm depending on the relative amount of Cd precursor employed in the synthesis, with PLQY as high as 17% at 1426 nm (Figure 1b and Table 1). Such PLQY was obtained without any postsynthesis treatment of the QDs or overgrowth of a wide-bandgap shell material. In stark contrast, the InAs QDs featured a PLQY below the instrument detection limit, and their PL spectrum, acquired using a high-power CW laser at 532 nm, peaked at 1050 nm and had a broad below-bandgap contribution most likely due to trap emission that was absent in the In­(Cd)As QDs (Figure 1b). In addition, the PL spectra of the In­(Cd)As QDs had full width at half-maximum (FWHM) values comparable to those reported for InAs@ZnSe QDs emitting at similar wavelengths (Table 1),^18^ further highlighting the beneficial role of Cd in improving the optical properties of InAs QDs. Based on powder X-ray diffraction (XRD), both InAs and In­(Cd)As QDs had the characteristic zinc-blende InAs structure (ICSD 98-002-4518) with no trace of secondary undesired byproducts (Figure 1c). TEM analysis revealed a systematic increase in QD size when increasing the Cd/In precursor ratio (Figures 1e–g and S3 and Table 1). These QDs retain the characteristic tetrahedral morphology commonly observed for amino-As-based InAs QDs, with occasional slight truncation at the corners.^22,31,32^ Elemental analyses, performed via inductively coupled plasma optical emission spectroscopy (ICP-OES), indicated that the amount of Cd present in the QDs increased from 0.17 to 0.35, expressed as the Cd/As atomic ratio, as the Cd/In precursor ratio was raised from 0.1 to 0.5 (Table 1). This was followed by a concomitant decrease in the In/As ratio, which was 1.17 in InAs QDs and decreased to 0.97 in samples made with progressively higher amounts of CdCl_2_ (Table 1).

**Figure 1:**
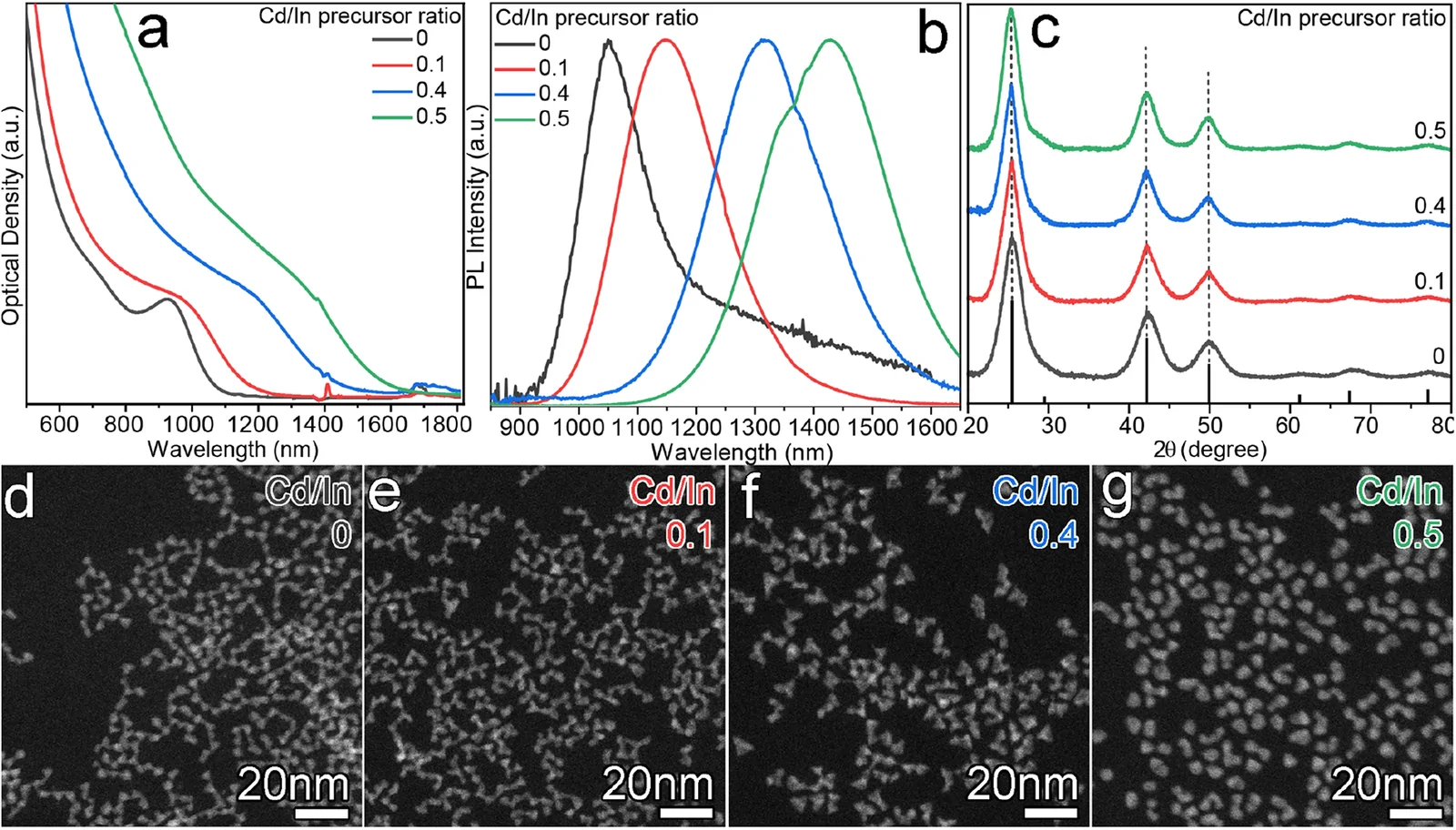
(a) Optical absorption spectra, (b) Normalized PL spectra, (c) XRD patterns, and HAADF-STEM images of (d) InAs and (e–g) In­(Cd)­As QDs synthesized with different Cd/In precursor ratios.

**Table 1:** Elemental Composition of InAs and In­(Cd)As QDs (Measured by ICP-OES), the Resulting QD Stoichiometry, Together with the Corresponding PL Peak Position, FWHM, PLQY, and Mean QD Size (Determined by TEM)

	elemental ratios in the QDs				PL analysis			
Cd/In precursor ratio	In/As	Cd/In	Cd/As	stoichiometry	PL peak (nm)	FWHM (meV)	PLQY (%)	QD size (nm)
0	1.17			In_1.17_As	1050		<1	3.4 ± 0.3
0.1	1.07	0.16	0.17	In_1.07_Cd_0.17_As	1147	185	21	3.5 ± 0.4
0.4	1.00	0.31	0.31	InCd_0.31_As	1319	161	15	3.9 ± 0.5
0.5	0.97	0.36	0.35	In_0.97_Cd_0.35_As	1426	150	17	4.5 ± 0.5

Overall, these data indicate that CdCl_2_ has an influence on the nucleation and growth kinetics of the InAs QDs, leading to (i) a redshift of the absorption and PL of the QDs with respect to pure InAs QDs of the same size; (ii) high PL efficiency with no need to perform any postsynthesis treatment. Regarding the first point, InAs QDs exhibiting absorption and PL features analogous to those of the In­(Cd)As QDs presented here are typically characterized by larger sizes. For example, InAs QDs reported by Panda et al., with sizes of 4.6 and 6.3 nm, featured PL peaks (upon ZnSe shelling) at 1160 and 1430 nm, respectively,^18^ whereas the In­(Cd)As QDs reported here exhibited PL at 1147 and 1426 nm for sizes of 3.5 and 4.5 nm, respectively (Figure S2i,j). For the second point, it is well documented that InAs QDs prepared with either amino-As or TMS-As do require a further postsynthesis treatment, namely, a shelling step comprising either CdSe, InP, or ZnSe overgrowth, in order to elicit PL.^16,33^ We hypothesize that the observed redshift and enhanced PL of In­(Cd)As QDs can be attributed to the incorporation of Cd either within the InAs QDs or at their surface, as recently observed for Zn atoms when employing ZnCl_2_ as an additive.^24^

To verify this hypothesis, we first performed high-resolution scanning transmission electron microscopy (HR-STEM) imaging combined with energy-dispersive X-ray spectroscopy (EDX) elemental mapping of In­(Cd)As QDs to probe the spatial distribution of Cd. The EDX maps showed a homogeneous distribution of In, As, and Cd atoms throughout the QDs (Figure 2a), indicating the presence of Cd in the QDs. However, the Cd signal was relatively low and insufficient to conclusively pinpoint whether Cd is inside the QD or primarily on its surface. We subsequently characterized the InAs and In­(Cd)As QDs by Raman spectroscopy. In the spectrum of the InAs QD sample, the peaks at 212 and 228 cm^–1^, corresponding to transverse optical (TO) and longitudinal optical (LO) modes of InAs, were identified (Figure 2b). These peaks shifted toward lower wavenumbers and were asymmetrically broadened in the In­(Cd)As QD samples (Figure 2b). The results were similar to those observed upon the insertion of exogenous metal cations into binary metal chalcogenide or oxide QDs (i.e., the formation of alloyed nanostructures), suggesting that in the present system Cd atoms are indeed inside the InAs structure, that is, an In–Cd–As alloy is formed.^34–36^ This interpretation is further supported by the evidence that no changes in the Raman modes of InAs QDs are observed when only surface In atoms are replaced by Zn, as shown in a previous work from our group,^25^ or by Cd atoms (*vide infra*). It is worth highlighting that such alloys are not reported in the bulk phase and might form as metastable structures at the nanoscale.

**Figure 2:**
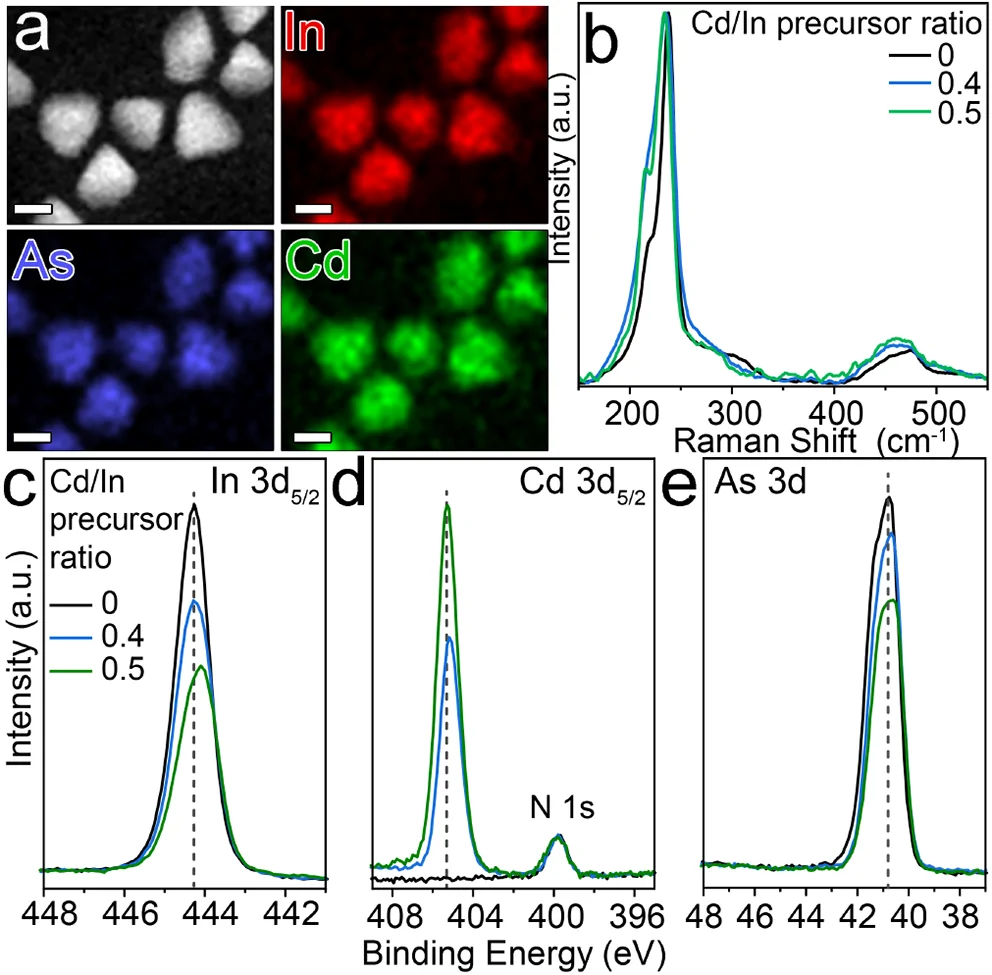
(a) STEM image and corresponding EDX elemental maps of In­(Cd)­As QDs prepared with a Cd/In precursor ratio of 0.5. Scale bars correspond to 5 nm. (b) Raman spectra of InAs and In­(Cd)As QDs. High-resolution X-ray photoelectron spectroscopy (XPS) spectra showing (c) In 3d_5/2_, (d) Cd 3d_5/2_, and (e) As 3d signals collected on InAs and In­(Cd)As QDs. Close to the Cd peak, the N 1s signal is ascribed to the surface oleylamine.

X-ray photoelectron spectroscopy (XPS) data were in agreement with ICP elemental analysis, confirming the presence of Cd in In­(Cd)­As QDs (Table S3). In­(Cd)As QDs exhibited the characteristic Cd 3d doublet, with the Cd 3d_5/2_ component located at a binding energy (BE) of (405.1 ± 0.2) eV for the sample prepared with Cd/In = 0.4 and slightly shifted toward higher BE (405.3 ± 0.2) eV for the sample prepared with Cd/In = 0.50, that is, with higher Cd content. The increase in Cd content in the In­(Cd)As QDs corresponded also to a systematic shift toward lower BE and broadening of both the In and As core signals: the In 3d_5/2_ peak shifted from 444.3 to 444.2 eV, with the FWHM going from 0.9 to 1.1 eV, and the As 3d_5/2_ signal shifted from 40.7 to 40.5 eV, with the FWHM slightly increasing from 0.85 to 0.95 eV, as the composition changed from pure InAs to In­(Cd)As using a Cd/In precursor ratio of 0.50 (Figure 2c–e). Overall, our XPS data indicate that (i) Cd is present in the QDs not in the form of CdCl_2_ (Z-type ligand), as the Cd 3d_5/2_ BE of such a compound was measured to be 406.6 eV (Figure S4); (ii) the In and As core levels exhibit small but systematic shifts toward lower BE with increasing Cd content. Although the magnitude of these shifts (0.1–0.2 eV) is comparable to the uncertainty associated with absolute BE determination (±0.2 eV) and therefore cannot, by itself, be regarded as conclusive evidence of alloy formation, the shifts are accompanied by a systematic broadening of both the In and As signals, consistent with an increased heterogeneity of their local chemical environments. Taken together, these XPS observations are consistent with a perturbation of the internal In–As environment upon Cd incorporation and corroborate the indication from Raman analysis of the formation of In–Cd–As alloys.

To further corroborate the formation of In–Cd–As alloyed QDs and to exclude the possibility that Cd atoms are solely located on the surface of InAs QDs, we also performed a control experiment in which InAs QDs were postsynthesis treated with CdCl_2_. This treatment was intended to promote the preferential incorporation of Cd atoms on the surface of InAs QDs, and the resulting sample is named “Cd-InAs QDs” hereafter. Previous studies on postsynthesis treatments on colloidal QDs, and in particular cation exchange reactions, have shown that replacement of host cations of preformed nanocrystals (In in the present case) with new guest cations (Cd in the present case) is thermally assisted, especially in III–V systems, and, when performed at the mild temperatures employed in the present work (i.e., 130 °C), is generally limited to the surface of the nanocrystals.^24,30,37,38^ The postsynthesis treatment that we developed, carried out at 130 °C, did not alter the size of the QDs (Figure S5) and led to the incorporation of Cd onto the InAs QDs, most likely only on their surface. Indeed, ICP-OES and quantitative XPS analyses revealed a Cd/As atomic ratio of 0.18 in the resulting QDs (Tables S2 and S3), a value comparable to that measured for the In­(Cd)As QDs made with a Cd/In precursor ratio of 0.1. The optical absorption of the Cd-InAs QDs was slightly red-shifted compared to the InAs sample prior to the CdCl_2_ treatment, but their PL stayed low (Figure 3a). The Raman analysis of the Cd-InAs QD sample revealed that the TO and LO modes were neither shifted nor broadened compared to those of the starting InAs sample (Figure 3b), in stark contrast with the In­(Cd)As samples discussed above. The XPS analysis of Cd-InAs QDs indicated the effective incorporation of Cd atoms (with Cd 3d_5/2_ BE being 405.1 ± 0.2 eV), with no presence of Cd in the form of CdCl_2_ (Figure 3d). Contrary to what was observed in the In­(Cd)As QD cases, the postsynthesis incorporation of Cd did not lead to any shift in the BE of In and As signals (Figure 3c,e). Together with XPS, Raman, and optical data for the In­(Cd)As QDs, these additional results provide further evidence that the use of CdCl_2_ as an additive in the synthesis of InAs QDs results in the formation of alloyed In–Cd–As QDs, whereas its use in the postsynthetic treatment of InAs QDs results in a Cd-for-In substitution that is mainly limited to the QD surface.

**Figure 3:**
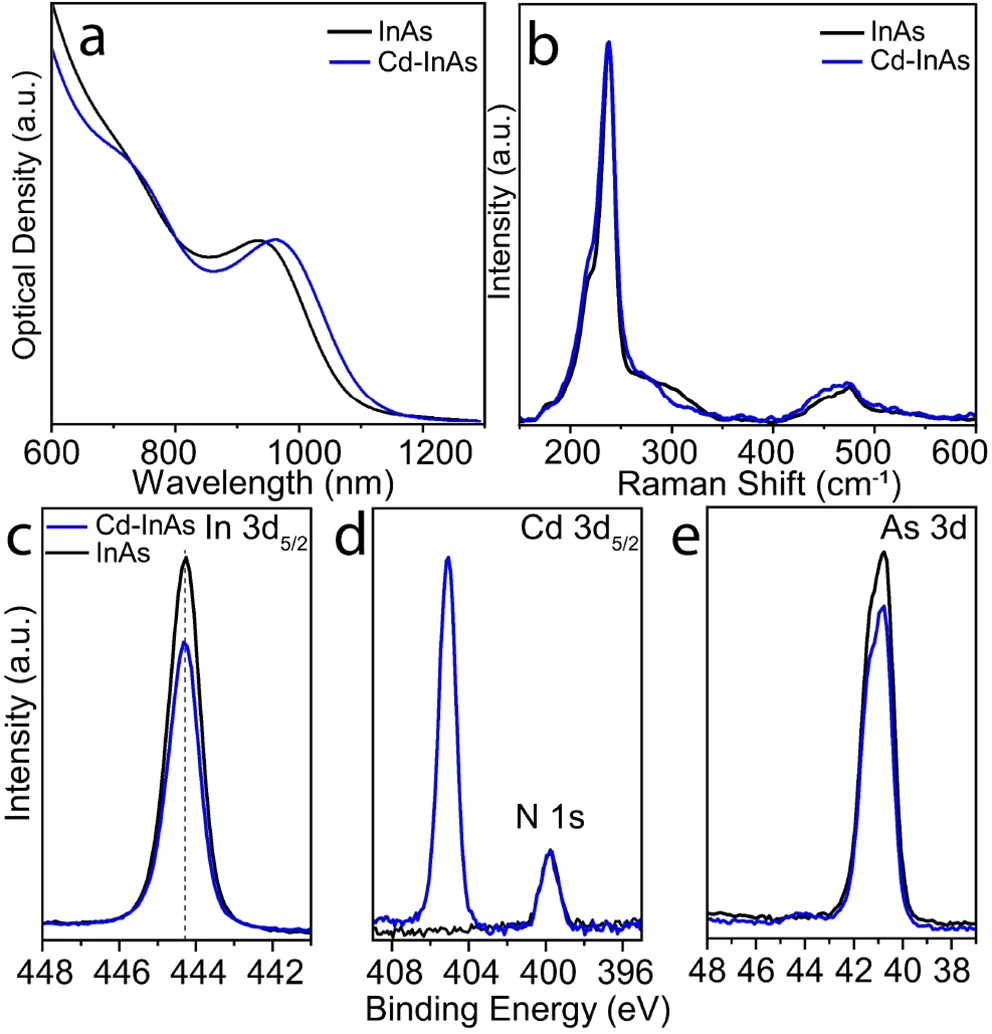
(a) Optical absorption spectra, (b) Raman spectra, and (c–e) XPS spectra of InAs and Cd-InAs QDs.

Having established the structural properties of the In­(Cd)As QDs, we then carried out time-resolved photoluminescence (TRPL) and transient absorption spectroscopy (TAS) to gain insights into the optical properties of these newly synthesized systems. Figure 4a reports the PL decay curves of In­(Cd)­As. While for InAs QDs the PL lifetime fell close to the instrument temporal resolution (∼1 ns, see Figure S6) due to the typical fast nonradiative decay associated with trapping in these systems,^24^ In­(Cd)As QDs showed a slow ns-long signal. Specifically, the samples synthesized with Cd/In precursor ratios of 0.1 and 0.4 had an initial decay component of ∼10 ns, accounting for approximately 35% of the signal, followed by a slower ∼60 ns component. In the sample synthesized with a Cd/In precursor ratio of 0.5, the latter became the sole decay component. Despite the monoexponential PL decay of such a sample, its PLQY reached only 17%. To better understand this apparent contrast, we combined the PLQY with the PL recombination rate (*k*_PL_) of the three different In­(Cd)As samples, and we retrieved the expected linear scaling of their radiative recombination rate (*k*_rad_ = *k*_PL_ × PLQY) with the PL energy (Figure 4b), which yielded radiative lifetimes (τ_rad_ = 1/*k*_rad_) that progressively increased from ∼200 to ∼400 ns as the bandgap of the QDs decreased. These results suggest that both the PLQY and the PL lifetime of In­(Cd)As QDs are most likely limited by dynamic interactions with the lattice via strong exciton-phonon coupling, a very efficient process in infrared-emitting materials, or ultrafast trapping events due to a homogeneous population of defects.

**Figure 4:**
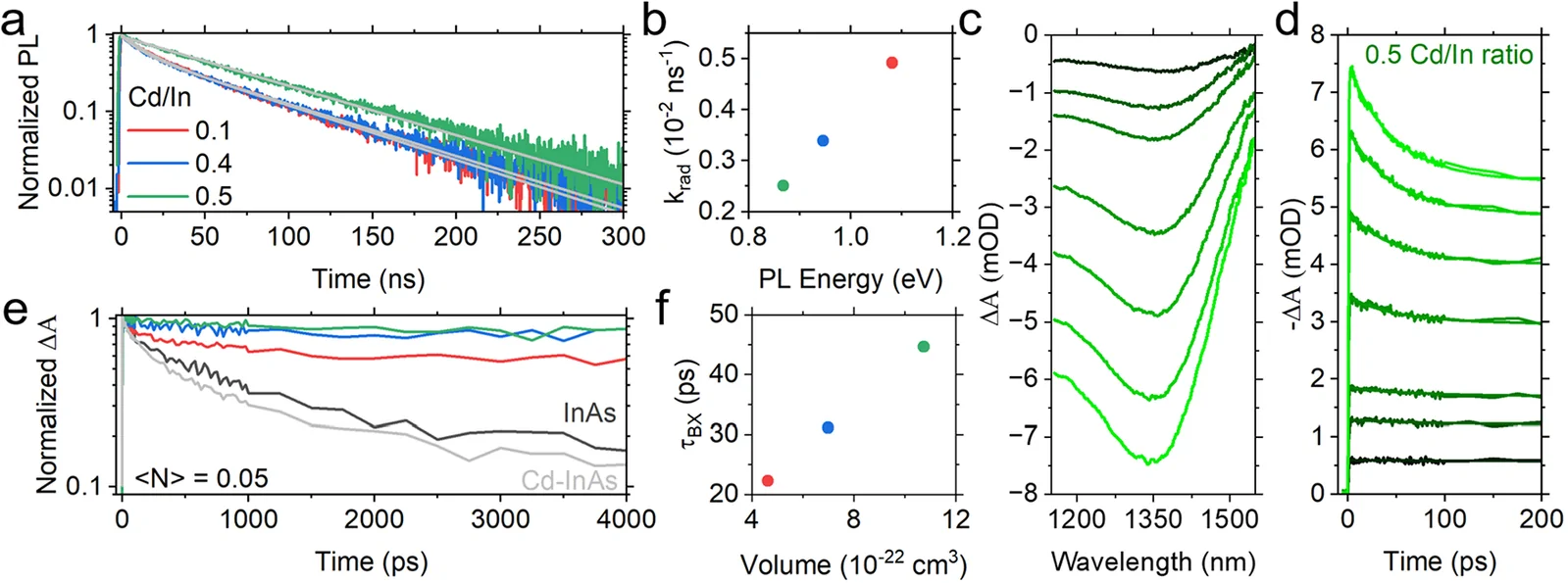
Time-resolved PL and TAS of In­(Cd)As QDs. (a) PL decay curves of InAs and In­(Cd)As QDs and relative fit (gray solid lines). (b) Radiative recombination rate vs PL energy. (c) Δ*A* spectra at 5 ps and (d) Δ*A* decay at the maximum bleach wavelength (1350 nm), and relative fit, increasing the pump fluence to generate ⟨*N*⟩ = 0.05, 0.1, 0.2, 0.4, 0.6, 0.8, and 0.9 for In­(Cd)As QDs synthesized with a Cd/In precursor ratio of 0.5. (e) Comparison of the bleach decay of the In­(Cd)As QDs with those of InAs and Cd-InAs QDs (light gray curve) for ⟨*N*⟩ = 0.05. All samples were excited at 800 nm. (f) Volume scaling of the biexciton lifetime of In­(Cd)As QDs.

To further investigate the charge carrier losses that might limit the exciton PL efficiency on an ultrafast time scale and gain insights into the multiexciton dynamics, we performed TAS on all investigated systems. At low fluence, InAs QDs evidenced a clear decay of the bleach due to exciton trapping (Figure 4e), in agreement with the low PLQY and fast PL decay. Notably, this process was progressively inhibited by increasing the amount of Cd, with the samples made with Cd/In precursor ratios of 0.4 and 0.5 showing a constant bleach signal up to 4 ns (Figure 4e). This aspect highlights that the PLQY in In­(Cd)As QDs is most likely strongly limited by exciton-phonon coupling rather than ultrafast trapping. Another important finding was that Cd-treated InAs QDs exhibited a bleach decay comparable to that of the pristine InAs QDs (Figure 4e). In both cases, the dynamics were well described by an initial ∼100 ps component followed by a ∼1 ns decay (Figure S6 and Table S4). This observation further highlights that the mere incorporation of Cd on the QD surface is insufficient to produce the substantial changes in the optical properties observed for In­(Cd)As QDs.

To gain insights into the biexciton Auger recombination of In­(Cd)­As QDs, we performed TAS at increasing pump fluences, generating an average number of excitons ⟨*N*⟩ ranging from ∼0.05 to 0.9. Figure 4c,d shows the results of this experiment for the In­(Cd)­As sample made with a Cd/In precursor ratio of 0.5 as a representative example. The Δ*A* spectra at 5 ps (Figure 4c) were characterized by the enhancement of the bleach in correspondence with the first absorption transition of the QDs, due to progressive charge carrier occupation of the band edge. Looking at the bleach kinetics in Figure 4d, by increasing the exciton population, a fast component associated with biexciton Auger recombination emerges on top of the slower decay due to single excitons, which persists up to 4 ns. To quantify the recombination lifetime of the biexciton (τ_BX_), we performed a global fit of the kinetics for ⟨*N*⟩ < 1 (Figures 4d and S7), corresponding to the regime where the multiexciton decay is dominated by biexcitons for Poisson statistics, using the function^39^

$$\Delta A\left(t\right)={\Delta A}_{X,\infty }+{\Delta A}_{BX}e^{-k_{BX}t}$$

Here, *k*_BX_ is kept as a global fit parameter and corresponds to the biexciton decay rate due to Auger recombination (*k*_BX_ = 1/τ_BX_), while Δ*A*_X,∞_ accounts for the remaining persistent signal due to the band-edge population of single excitons. As shown in Figure 4f, τ_BX_ increased with increasing Cd/In precursor ratio, with the In­(Cd)As QDs synthesized using a Cd/In precursor ratio of 0.5 showing a τ_BX_ value of ∼50 ps. The increase in the biexciton lifetime with increasing Cd/In precursor ratio was rationalized in terms of the slight increase in the size of the corresponding In­(Cd)As QDs, which showed the expected linear scaling with the nanocrystal volume.^40,41^

To rationalize our findings and to elucidate how Cd atoms possibly modulate the electronic structure of InAs QDs when inserted either in their lattice or on their surface, we performed DFT calculations. These were made on a ∼3 nm tetrahedral InAs model exhibiting (100), (111), and (−1–1–1) facets, passivated with chlorine and methylamines (the latter mimicking the use of oleylamine in the synthesis). The model had no surface reconstruction to evaluate whether Cd inclusion had effects on the trap states associated with undercoordinated surface atoms (Figure 5a­(iii)). To comprehensively evaluate this electronic landscape, we tracked four complementary quantum-mechanical descriptors: (i) the projected density of states (PDOS), which describes the direct contribution of each element to each individual molecular orbital (MO); (ii) the crystal orbital overlap population (COOP),^42^ which describes the bonding characters (i.e., antibonding, nonbonding and bonding) (Figure S8); (iii) the inverse participation ratio (IPR),^42^ which serves as a metric for wave function localization (Figure S8); and (iv) the charge-flow analysis^27^ (Figure S9), which maps the direction and magnitude of the electron density flow within the QD models.

**Figure 5:**
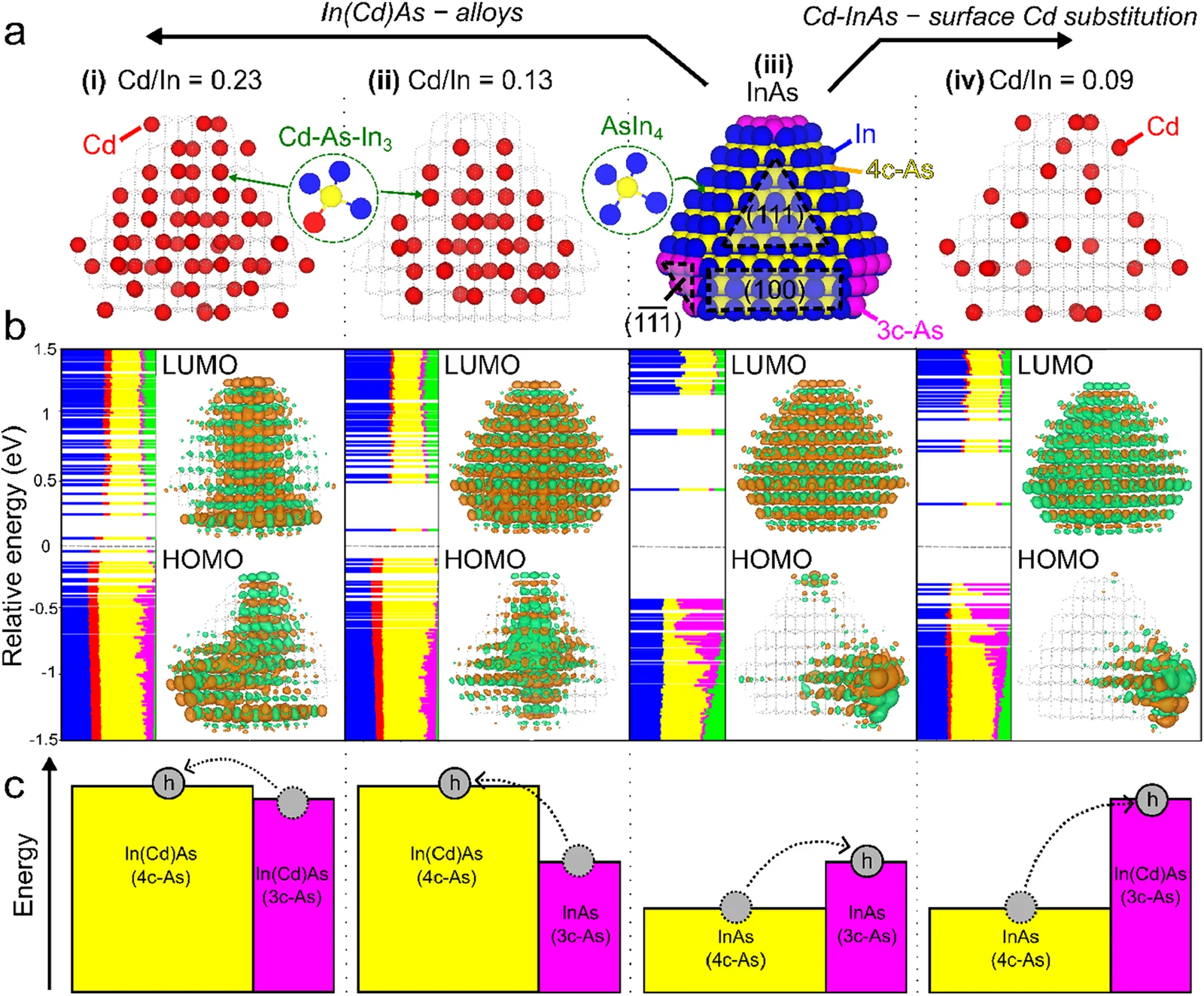
(a) Atomistic models of ∼3 nm tetrahedral QDs: In­(Cd)­As alloyed QDs with Cd/In atomic ratios of (i) 0.23 and (ii) 0.13; (iii) InAs QD; (iv) surface-substituted Cd-InAs QD with a final Cd/In atomic ratio of 0.09. Color code: In = blue, Cd = red, 4-coordinated bulk-like As (4c-As) = yellow, 3-coordinated surface As (3c-As) = magenta. For simplicity, ligands were omitted in the structural models. (b) PDOS plots (same color code as panel (a), including Cl in green and CH_3_NH_2_ in gray) along with their corresponding HOMO and LUMO wave functions; Horizontal gray dashed lines mark the Fermi level, above (below) which MOs are unoccupied (occupied) and are set at the energy origin. (c) Schematic energy diagrams outlining the hole (h) localization mechanism at the VBM. The diagrams illustrate how internal Cd alloying drives the hole into the QD core (4c-As), whereas surface Cd substitution traps the hole at the QD surface (3c-As).

In agreement with previous works,^24–27^ the pristine InAs QDs are characterized by a dense manifold of trap states, localized right above the valence band maximum (VBM), originating from 3-coordinated (3c) As atoms at the anion-rich (−1–1–1) facets (Figure 5b­(iii),c­(iii)). From this pristine baseline, we explored two structurally distinct scenarios that mirror our experiments: In­(Cd)As alloys (Figure 5a­(i,ii)) and surface Cd substitution on InAs QDs (Figure 5a­(iv)). In the case of alloys, we progressively substituted In with Cd atoms from the core toward the surface subshells (Figure 5a­(i,ii)) and obtained In­(Cd)As models featuring Cd/In atomic ratios of 0.13 and 0.23. These models are qualitatively similar to those obtained experimentally (Table 1), where each substituted Cd forms part of a local Cd–As–In_3_ tetrahedron with a 4-coordinated (4c) As atom at its center (Figure 5a­(i,ii) inset). For the surface Cd substitution, the Cd-for-In substitution was restricted solely to the surface subshell and maintained consistent, local Cd–As–In_3_ environments at the surface. Henceforth, in Figure 5a­(iv), this surface-substituted QD model will be named Cd-InAs.

Additionally, we assessed the thermodynamic feasibility of these configurations by calculating the QD formation energy (Δ*E*_f_) from its components: InAs monomers, InCl_3_ and CdCl_2_ units (see explanation in the SI and Tables S5 and S6). We found that the Cd^2+^ to In^3+^ substitution is energetically favorable for both In­(Cd)As and Cd-InAs cases. First, the formation of In­(Cd)­As alloys is in all cases exergonic, featuring a Δ*E*_f_ of –8 kcal·mol^–1^ per CdCl_2_ in the Cd/In = 0.13 case and becoming less negative (−2 kcal·mol^–1^ per CdCl_2_) at a ratio of Cd/In = 0.23. This also indicates that further incorporations become energetically disfavored. Second, the formation of Cd-InAs QDs, namely the substitution of surface In^3+^ by Cd^2+^ at the QD surface, is also highly favorable, yielding a Δ*E*_f_ of −17 kcal·mol^–1^ per CdCl_2_ at Cd/In = 0.09. Ultimately, these data demonstrate a clear trend toward Cd incorporation into the lattice at low Cd/In ratios (up to 0.23); while this process slightly favors the surface, core incorporation remains energetically viable. It is worth highlighting that, consistent with the absence of reported bulk In–Cd–As alloys, the Cd-for-In substitution in bulk InAs was calculated to be energetically unfavorable (Figures S10 and S11). This was attributed to the absence of the surface degrees of freedom required to compensate for the resulting Cd^2+^ → In^3+^ charge imbalance. Regarding the electronic structure of these systems, by using the midgap energy as a common visual plotting origin to track the overall bandgap evolution, we observe that Cd alloying induces a systematic bandgap contraction (Figure 5b­(i–iv)-PDOS). More importantly, the magnitude of this contraction depends heavily on whether Cd atoms are placed in the core or strictly on the surface of the InAs QDs. In all cases studied here (Figure 5), the bandgap contraction closely correlates with the optical redshifts observed experimentally (Figures 1a and 3a). Since Cd^2+^ and In^3+^ ions differ appreciably in ionic radius, Cd incorporation might be expected to strain the host lattice and thereby contribute to modifying the bandgap. On the other hand, we observed that interatomic distances in In­(Cd)As alloys remain essentially unchanged from those of pristine InAs in all models (Figure S12). This result is in line with the absence of any measurable shift in the XRD patterns of InAs QDs following Cd incorporation (Figure 1c). Hence, Cd substitution can be considered isostructural, and strain can be ruled out as a source of the bandgap redshift. Of particular interest, in the In­(Cd)As models, the physical origin of this redshift is elucidated by the COOP analysis (Figure S8). The introduction of Cd fundamentally alters the local In–As bonding character; specifically, the formation of local Cd–As–In_3_ environments in the core region weakens the In–As bonds, leading to a significantly less bonding character at the VB. Conversely, the antibonding character of the In–As bond at the conduction band minimum (CBM) remains largely unaffected. Because the VB states are destabilized considerably more than the CB states, we can deduce that the observed bandgap contraction is highly asymmetric, driven by a more pronounced upward shift of the VBM. This asymmetric shift is further corroborated on an absolute energy scale using a noninteracting spectator H_2_ molecule as a reference (Table S7). Consequently, this upward shift of the VBM is accompanied by a rise in energy of 4c-As bulk-like MOs, causing the deep 3c-As surface trap states to become energetically shallow (Figures 5b­(i,ii)-PDOS, 5c­(i,ii) and S8), which explains the increase in PLQY and the suppression of ultrafast trapping experimentally observed both by time-resolved PL and transient absorption spectroscopy in In­(Cd)­As QDs. Crucially, this fundamental weakening of the In–As bonds provides a direct microscopic explanation for our XPS measurements. As the In–As interactions become less bonding upon Cd incorporation, the atoms in the core region are less tightly bound. This localized destabilization naturally translates to a reduction in the energy required to eject core electrons; indeed, as the Cd content increases in the alloys, both the In 3d_5/2_ and As 3d peaks systematically shift to lower binding energies (Figure 2c–e), mirroring the bond-weakening mechanism extracted from the COOP analysis (Figure S8). On the other hand, in the surface-substituted Cd-InAs case, our models predict a bandgap contraction that is significantly less pronounced than in the fully alloyed systems. This modest bandgap narrowing aligns with the slight optical redshift observed experimentally after the CdCl_2_ postsynthesis treatment (Figure 3a) and the absence of sizable differences in the charge carrier dynamics and PLQY of Cd-InAs and pristine InAs. Importantly, as confirmed by our COOP analysis (Figure S8), the internal AsIn_4_ environment in the QD core region is unaffected by the Cd-for-In substitution. Since these internal In–As bonds at the VB retain their bonding character, the 4c-As MOs are not pushed to higher energies relative to the midgap. Consequently, the 3c-As surface trap states fail to become shallow (Figures 5b­(iv)-PDOS and S8). Instead, they remain deep and localized within the bandgap (Figures 5c­(iv) and S8), similarly to the pristine InAs QDs (Figure 5c­(iii)), which explains the lack of PL efficiency enhancement observed experimentally (Figure 3a). We also calculated the effects of CdCl_2_ binding (as Z-type ligands) to undercoordinated As atoms on the (−1–1–1) facets and found that this configuration was ineffective in suppressing nonradiative trap states (Figure S13). This further excludes surface-bound Cd species as being able to enhance the PLQY of InAs QDs. Furthermore, the high preservation of the bonding (antibonding) character of the In–As bonds at the VB (CB) is consistent with the XPS data for the Cd-InAs QDs (Figure 3c−e). Because the internal In–As bonds preserve their bonding nature, the energy required to eject their core electrons remains largely unchanged, resulting in no shift to lower binding energies for the host In and As core levels.

Overall, our DFT calculations indicated that Cd incorporation in InAs QDs, thus forming In–Cd–As alloys, leads to a narrowing of their bandgap and causes the deep 3c-As surface trap states to become shallow, so that they are “swept under the carpet” of the upward-shifted 4c-As MOs. Such features align with the observed red-shifted absorption and efficient PL of our In­(Cd)As QDs, further supporting the conclusion that these QDs feature an In–Cd–As alloyed composition. DFT results also revealed that a Cd-for-In substitution limited strictly to the surface of InAs QDs produces only a slight bandgap narrowing and fails to replicate the PLQY enhancement achieved in the alloyed case, consistent with the behavior of our Cd–InAs QDs.

## Conclusion

In this work, we investigated the effect of CdCl_2_ employed either as a synthesis additive for amino-As-based InAs QDs or as a postsynthesis treatment of preformed InAs QDs. When employed as a synthesis additive, CdCl_2_ promoted the formation of alloyed In­(Cd)As QDs, incorporating up to 30% Cd into the QDs. Compared to pure InAs QDs, Cd incorporation induced bandgap narrowing, resulting in red-shifted absorption, and significantly enhanced the PL efficiency and lifetime. By employing various amounts of CdCl_2_, it was possible to synthesize In­(Cd)As QDs through a one-pot procedure with absorption spectra tunable from ∼950 to ∼1400 nm and PLQY as high as 17% at ∼1426 nm, without the need for postsynthesis shell growth. Conversely, CdCl_2_ post-treatment of preformed InAs QDs resulted only in surface Cd incorporation (Cd-InAs), producing a modest redshift and no significant PL enhancement. Transient absorption spectroscopy confirmed these differences, showing severe ultrafast trapping in both InAs and Cd-InAs QDs, whereas In­(Cd)As QDs exhibited suppressed trapping pathways, persistent single-exciton bleach, and biexciton lifetimes scaling with particle volume.

DFT calculations rationalized these observations, indicating that although the In­(Cd)As alloy formation is unfavorable in the bulk, it becomes feasible at the nanoscale. Cd incorporation weakens core In–As bonds, inducing an upward shift of the VBM that narrows the bandgap and shifts surface trap states closer to the valence band edge, thereby enhancing PLQY. In contrast, Cd incorporation limited to the surface of InAs QDs preserves the core In–As bonding character, preventing VBM modulation and leaving deep trap states unaffected, thus explaining the absence of PL enhancement. Overall, this work demonstrates that metal-halide-mediated alloying strategies provide a powerful route toward next-generation infrared-emitting InAs-based QDs.

## Materials and Methods

### Materials

Aluminum­(III) chloride (AlCl_3_, 99.999%, Sigma-Aldrich), lithium aluminum hydride (LiAlH_4_, 95%, Sigma-Aldrich), indium­(III) chloride (InCl_3_, 99.999%, Sigma-Aldrich), cadmium­(II) chloride (CdCl_2_, 99.99%, Sigma-Aldrich), tris­(dimethylamino)­arsine (amino-As, 99%, Strem), trioctylamine (TOA, 98%, Sigma-Aldrich), octadecene (ODE, 90%, Sigma-Aldrich), oleylamine (Olam, 70%, Sigma-Aldrich), pentane (anhydrous, >99%, Sigma-Aldrich), toluene (anhydrous, 99.8%, Sigma-Aldrich), tetrachloroethylene (TCE, anhydrous, 99%, Sigma-Aldrich), ethanol (anhydrous, 99.8%, Sigma-Aldrich). All the chemicals were used without further purification. ODE, Olam, and TOA were degassed at 120 °C under vacuum for 1.5 h before use.

### Preparation of Amino-As Precursor

0.2 mmol of amino-As was added to 0.5 mL of degassed Olam (resulting in a 0.4 M solution) in a vial, inside a N_2_-filled glovebox, and heated at 60 °C for 15 min.

### Synthesis of InAs QDs

The reducing agent TOA-AlH_3_ and InAs QDs were synthesized by following the procedure reported by Panda et al.^18^ In detail, 0.2 mmol of InCl_3_ was mixed with 3.3 mL of Olam and 6.7 mL of ODE in a 50 mL three-neck round-bottom flask inside the glovebox. The flask was then connected to a Schlenk line, avoiding exposure to air, and degassed for 1 h at 120 °C. The flask atmosphere was switched to nitrogen, and the temperature was raised to 240 °C. The amino-As precursor solution was injected at 240 °C, resulting in a slight drop in temperature (∼5 °C). After the temperature recovered (∼20 s), 0.8 mmol of TOA-AlH_3_ dissolved in 0.5 mL of ODE was swiftly injected, and the temperature was raised to 280 °C at a rate of 4 °C/min. The reaction was maintained at 280 °C for 5–40 min, followed by quenching with compressed air flow. 10 mL toluene was added upon cooling to 90 °C. The crude solution was centrifuged at 3000 rpm for 5 min, and the precipitate was discarded. Upon addition of ethanol to the supernatant, the resulting mixture was centrifuged at 6000 rpm for 5 min to isolate the QDs. The washing was repeated one more time, and the final precipitate was redispersed in TCE and stored in a glovebox for further use.

### Synthesis of In­(Cd)As QDs

In­(Cd)As QDs were synthesized using the same procedure described for InAs QDs, with the only modification being the addition of the desired amount of CdCl_2_. CdCl_2_ with Cd/In ratios of 0.1, 0.4, and 0.5 (0.02, 0.08, and 0.1 mmol, respectively) was added to the reaction mixture while keeping 0.2 mmol of InCl_3_ precursor fixed all the time. The injection of amino-As and TOA-AlH_3_ was performed at 240 °C, followed by heating to 280 °C for 5 min.

### CdCl_2_ Postsynthesis Treatment

For postsynthesis treatment with CdCl_2_, InAs QDs were first synthesized by the standard procedure, washed twice with ethanol, and redispersed in 3 mL of Olam (total ∼0.18 mmol In from ICP-OES). The dispersion was divided into two equal portions. In a typical post-treatment, one portion of the InAs QD dispersion was loaded into a three-neck flask, followed by the addition of 0.23 mL of a 0.2 M CdCl_2_–oleylamine precursor solution and 3.3 mL of ODE. The Cd/In precursor ratio corresponds to 0.5. The reaction mixture was degassed at room temperature for 10 min, and subsequently heated to 130 °C for 40 min. The QDs were washed twice with ethanol, redispersed in TCE, and stored in a glovebox.

### X-ray Diffraction (XRD)

XRD patterns were acquired using a Malvern-PANalytical Empyrean X-ray diffractometer equipped with a 1.8 kW Cu Kα sealed ceramic X-ray tube with microbeam collimators and a Pixcel3D^2×2^ area detector, operating at 45 kV and 40 mA. Concentrated QD solutions were drop-cast onto a silicon zero-diffraction single-crystal substrate for XRD measurement. A parallel-beam geometry in symmetric reflection mode was used to record X-ray diffraction patterns under ambient conditions. HighScore Plus software (PANalytical) was used for XRD data analysis.

### Transmission Electron Microscopy (TEM)

Diluted QD solutions were drop-cast onto ultrathin carbon-coated copper TEM grids. Bright-field TEM images were acquired on a JEOL JEM-1011 microscope with a thermionic source (W filament) operated at an acceleration voltage of 100 kV. High-resolution (HR) scanning transmission electron microscopy (STEM) images were acquired on a probe-corrected ThermoFisher Spectra 30–300 (S)­TEM operated at 300 kV. Images were acquired on a high-angle annular dark field (HAADF) detector with a current of ≈50 pA. Compositional maps were acquired using Velox, with a probe current of ≈150 pA and rapid rastered scanning of the beam. The energy-dispersive X-ray (EDX) signal was collected by a Dual-X system, comprising two detectors, one on either side of the sample, for a total acquisition solid angle of 1.76 sr.

### Inductively Coupled Plasma (ICP)

The elemental analysis was performed by inductively coupled plasma optical emission spectroscopy (ICP-OES) with an iCAP 6300 DUO ICP-OES spectrometer (Thermo Scientific). The samples were digested in 0.5 mL of aqua regia (HCl/HNO_3_ = 3/1 (v/v)) overnight and then diluted with 9.5 mL of Milli-Q water for measurements. The ICP-OES elemental analysis was affected by a systematic error of ∼5%.

### Raman Spectroscopy

Raman spectroscopy measurements were performed with a microRaman Invia 1000 (Renishaw, U.K.) using an excitation wavelength of 633 nm, a grating with 1800 lines/mm, and an incident power of 1 mW. An exposure time of 15 s was used for each spectrum, averaging at least 10 spectra for each sample. The samples were held in a nitrogen atmosphere inside a Linkam THMS600 chamber (Linkam Scientific, U.K.). The microscope was mounted with a Leica 50× long working distance objective. The data were analyzed using Renishaw Wire 5.6 software.

### X-ray Photoelectron Spectroscopy (XPS)

Highly concentrated QDs were drop-cast on highly oriented pyrolytic graphite substrates in a glovebox for XPS measurement. XPS measurements were carried out on a Kratos Axis Ultra^DLD^ spectrometer using a monochromatic Al Kα source, operated at 20 mA and 15 kV. High-resolution analyses were carried out at a pass energy of 20 eV, with an energy step of 0.1 eV, over a 300 × 700 μm^2^ area. Spectra were charge-corrected to the main line of the carbon 1s spectrum (due to C–C bonds) set to 284.8 eV. Spectra were analyzed using CasaXPS software (version 2.3.27).^29^

### Optical Measurements

Samples for optical measurements were prepared by diluting a portion of QD stock with 3 mL TCE or toluene in a 1 cm path length cuvette, inside the glovebox. The cuvettes were capped with airtight caps and sealed with parafilm before being taken out of the glovebox. The absorption spectra were recorded using a Varian Cary 5000 UV–vis–NIR spectrophotometer. All QD solutions were diluted to an optical density of ∼0.1 at 804 nm for PL and PLQY measurements. Steady-state PL and PLQY measurements were carried out using an Edinburgh Instruments FS5 equipped with a 150 W xenon arc lamp, excitation and emission monochromator, integrating sphere, and InGaAs-1650 NIR detector. An 804 nm CW laser was used as the excitation source for PLQY measurements. Time-resolved PL measurements were carried out using an Edinburgh Instruments FLS900 fluorescence spectrometer. The samples were excited with a pulsed laser diode (Edinburgh Instruments EPL-510, λ_ex_ = 508.2 nm, pulse width = 177.0 ps) and the emission was collected using an InGaAs detector coupled to a time-correlated single-photon counting (TCSPC) unit.

Transient absorption spectroscopy was carried out using a Harpia-TA system (Light Conversion), driven by a ytterbium-based laser (Pharos-SP-HP, Light Conversion) operating at 50 kHz with a temporal resolution of approximately 160 fs. The fundamental laser output beam, at 1030 nm, passes through a beamsplitter where 90% of the light seeds an optical parametric amplifier (Orpheus, Light Conversion) that generates the pump beam (800 nm) via nonlinear optical conversion. The remaining 10% is focused onto a YAG crystal, generating probe light covering the spectral window from 800 to 1600 nm. Before overlapping on the sample with the probe, the pump passed through a chopper operating at 75 Hz. The probe light is collected by a spectrometer (Andor Kymera 193i) using a silicon or an InGaAs detector coupled to a monochromator with a blaze at 800 nm and 150 grooves/mm.

### Computational Methodology

Atomistic simulations at the DFT level were carried out in vacuum with about 10 Å padding and a 400 Ry grid cutoff using the CP2K quantum chemical package.

Structural geometry optimizations and single-point electronic properties were performed using the high-local exchange HLE17 functional.^43,44^

The electronic structure was solved using the Gaussian and plane waves (GPW) method. Relativistic effects for all atoms were accounted for via Goedecker-Teter-Hutter (GTH) effective core potentials paired with a double-ζ valence polarized (DZVP) basis set.^45^

Unless otherwise noted, convergence followed CP2K defaults for geometry criteria; in practice, optimizations continued until the maximum residual force was at or below standard tight thresholds (≈10^–3^–10^–4^ Ha/bohr range). The geometry optimization was performed using the LBFGS algorithm. Wave function localization and bond characters were quantified via inverse participation ratio (IPR) and crystal orbital overlap population (COOP) workflows. Molecular orbital density surfaces were mapped using a constant wave function isosurface value of ±2.8 × 10^–3^ (e^–^/bohr^3^)^1/2^. The internal electronic redistribution induced upon chemical substitution was evaluated via charge-flow vector fields analysis.^27^

## Supplementary Material



## Notes

Synthesis optimization of In­(Cd)As QDs, optical absorption and emission spectra, XPS and ICP-OES elemental analysis data, size distribution histograms, XPS analysis of CdCl_2_, and additional spectroscopy and computational details (PDF)
