# Supplementary material for: Sweeping Surface Trap States under the Carpet: Cd2+ Inclusion in InAs Quantum Dots
Source: ACS Nano. 2026 Oct 30;20(44):30181–91. doi: 10.1021/acsnano.6c15005 (PMC13758395; doi:10.1021/acsnano.6c15005)
Supplement: Supplementary file 1 [file nn6c15005_si_001.pdf]

Supporting Information

Sweeping Surface Trap States Under the Carpet: Cd^{2+} Inclusion in InAs Quantum Dots

Habibul Arfin[a], Jordi Llusar*[a], Carmelita Rodà*[a], Satyaprakash Panda[a], Yurii P. Ivanov[b], Giorgio Divitini[b], Mirko Prato[c], Andrea Toma[d], Andrea Fratelli[a,e], Matteo Zaffalon[e], Sergio Brovelli*[e], Ivan Infante[f], Luca De Trizio*[g] and Liberato Manna*[a]

[a]Nanochemistry, [b]Electron Spectroscopy and Nanoscopy, [c]Materials Characterization, [d]Clean Room Facility and [g]Chemistry Facility, Istituto Italiano di Tecnologia, Via Morego 30, 16163 Genova, Italy

[e]Dipartimento di Scienza dei Materiali, Università degli Studi di Milano-Bicocca, Via R. Cozzi 55, 20125, Milano, Italy

[f]BCMaterials, Basque Center for Materials, Applications, and Nanostructures, UPV/EHU Science Park, Leioa 48940, Spain

S1. Optimization of the In(Cd)As QDs reaction conditions

First, we varied the reaction temperature while keeping the Cd/In ratio fixed to 0.5. **Figure S1** shows absorption spectra, photoluminescence (PL) and PL analysis data for QDs synthesized at different reaction temperatures. Increasing the reaction temperature leads to a progressive red-shift of both absorption and PL, consistent with faster growth and larger QD formation. By tuning the synthesis temperature, the PL peak can be adjusted from 850 nm to 1650 nm. Reaction carried out at 240- 280 °C (injection at 240 °C and growth at 280 °C) yielded QDs with narrow FWHM (**Figure S1j**) and high PLQY (**Figure S1k**). Elemental analyses performed via ICP-OES are reported in **Table S1**.

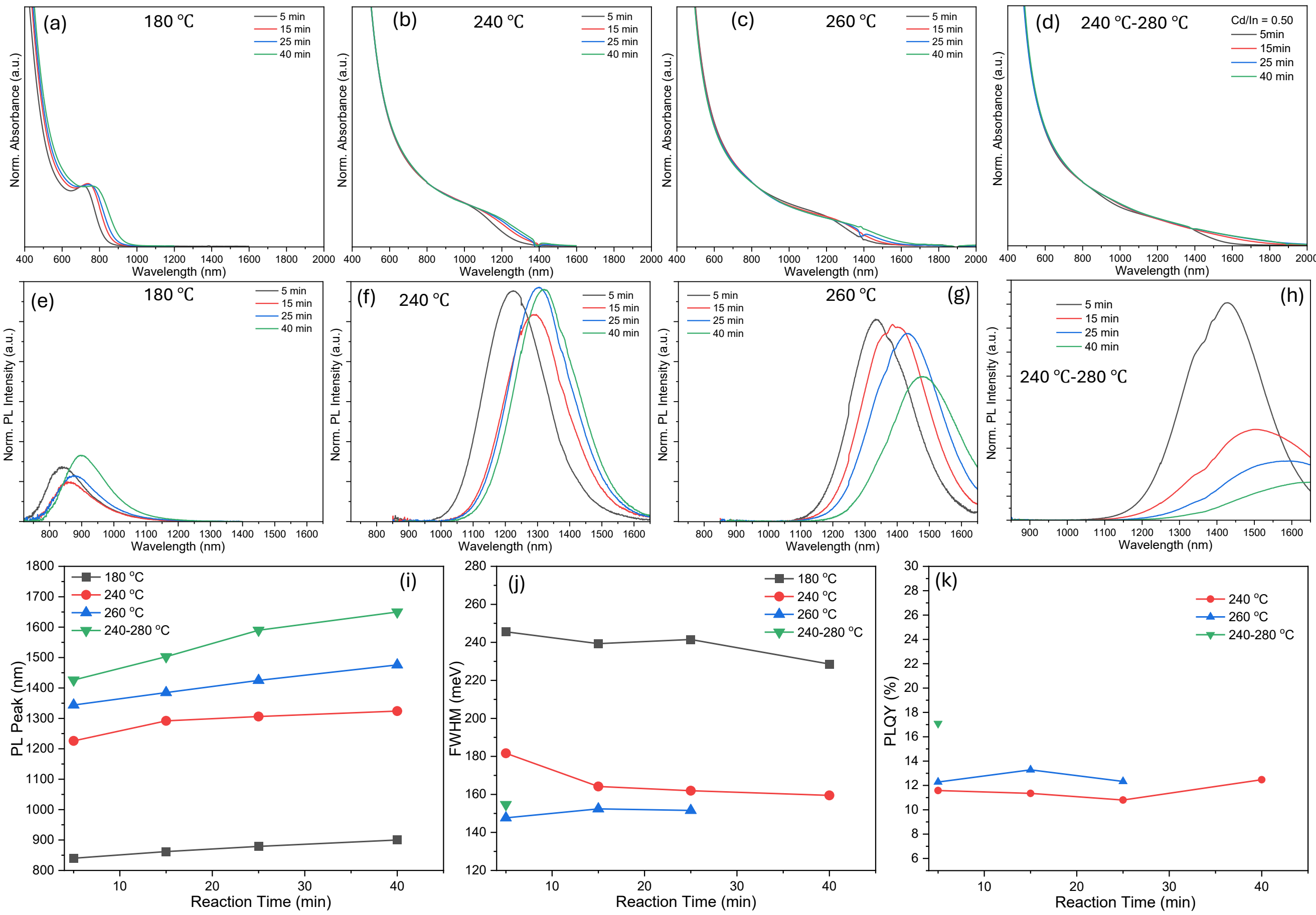

Figure S1. (a-d) absorption and (e-h) emission spectra, (i) PL peak position, (j) FWHM and (k) PLQY of In(Cd)As QDs synthesized at different temperatures with a Cd/In precursor ratio of 0.5. In (j-k) values for samples prepared at 240-280℃ for reaction times longer than 5 min are missing since the emission spectra extended beyond the spectrometer detection range, limiting the analysis. In panels e-h the PL was normalized to the absorption at the excitation wavelength (i.e. 700 nm).

Table S1. ICP-OES data of QDs synthesized at different temperatures with a Cd/In precursor ratio of 0.5

Reaction temperature	Reaction time	In/As	Cd/In	Cd/As
180 °C	5 min	1.11	0.30	0.34
	15 min	1.08	0.24	0.26
	25 min	1.11	0.28	0.32
	40 min	1.09	0.25	0.28
240 °C	5 min	1.04	0.26	0.28
	15 min	1.04	0.26	0.27
	25 min	1.02	0.26	0.26
	40 min	1.01	0.25	0.26
260 °C	5 min	1.03	0.32	0.33
	15 min	1	0.31	0.31
	25 min	1.02	0.29	0.3
	40 min	1.03	0.28	0.29
240-280 °C	5 min	0.97	0.36	0.35
	15 min	1.03	0.29	0.3
	25 min	1.03	0.28	0.29

	40 min	1.03	0.25	0.25

Then, we systematically varied the Cd/In precursor ratio from 0 to 1.0 at a fixed reaction temperature (injection at 240 °C and growth at 280 °C) and collected aliquots at 5, 15, 25 and 40 minutes. Higher Cd/In ratios lead to QDs with red-shifted absorption and emission, and for a fixed Cd/In ratio, both absorption and PL further red-shift with reaction time, as shown in **Figure S2**.

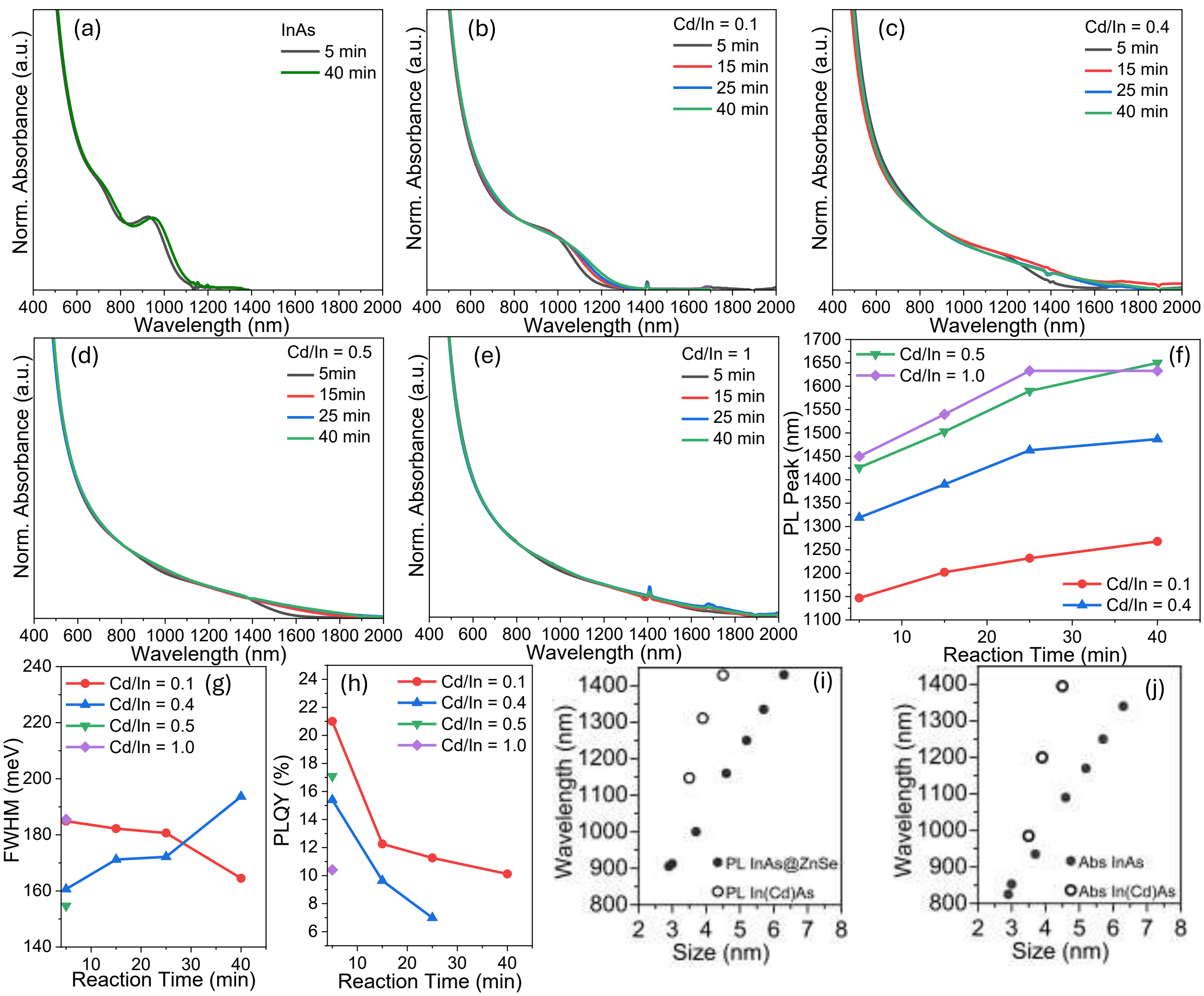

Figure S2. Absorption spectra of (a) InAs QDs and (b–e) In(Cd)As QDs synthesized with different Cd/In precursor ratios. (f) PL peak positions (g) PL FWHM and (h) PLQY of In(Cd)As QDs synthesized with different Cd/In precursor ratios. In panels (g) and (h), the FWHM and PLQY values beyond 5 min for samples prepared with Cd/In ratios of 0.5 and 1.0 are not reported since their PL spectra extended beyond the spectrometer's detection range (i.e. above 1650 nm), preventing reliable analysis. i) PL peak position Vs QD size of In(Cd)As and InAs@ZnSe QDs. j) Excitonic absorption peak position vs QD size of In(Cd)As and InAs QDs. PL peak positions of InAs@ZnSe QDs and InAs core sizes are taken from ref (1).

Table S2. ICP-OES data of QDs synthesized with different Cd/In precursor ratios at 240-280 °C (In(Cd)As QDs) and those prepared via post-synthesis treatment of InAs QDs with $CdCl_2$ (Cd-InAs)

Cd/In ratio used in reaction	Reaction time	In/As	Cd/In	Cd/As
0.0	5 min	1.17		
	40 min	1.17		
0.1	5 min	1.07	0.16	0.17
	15 min	1.06	0.15	0.16
	25 min	1.06	0.15	0.16
	40 min	1.05	0.16	0.16
0.4	5 min	1.00	0.31	0.31
	15 min	0.86	0.33	0.28
	25 min	1.01	0.26	0.26
	40 min	0.98	0.24	0.23
0.5	5 min	0.97	0.36	0.35
	15 min	1.03	0.29	0.30
	25 min	1.03	0.28	0.29
	40 min	1.03	0.25	0.25
1.0	5 min	0.97	0.32	0.31
	15 min	0.99	0.26	0.25
	25 min	1.00	0.23	0.23
	40 min	1.01	0.22	0.23
$CdCl_2$ Post-treated InAs		1.01	0.18	0.18

We selected 5 min as the reaction time because it was possible to obtain samples with emission within the spectrometer's detection range, minimal FWHM, and high PLQY. We excluded the sample made with a Cd/In precursor ratio of 1 since we did not observe any further inclusion of Cd (Table S2), nor further absorption or PL redshift compared to the sample made with a Cd/In precursor ratio of 0.5 (**Figure S2**).

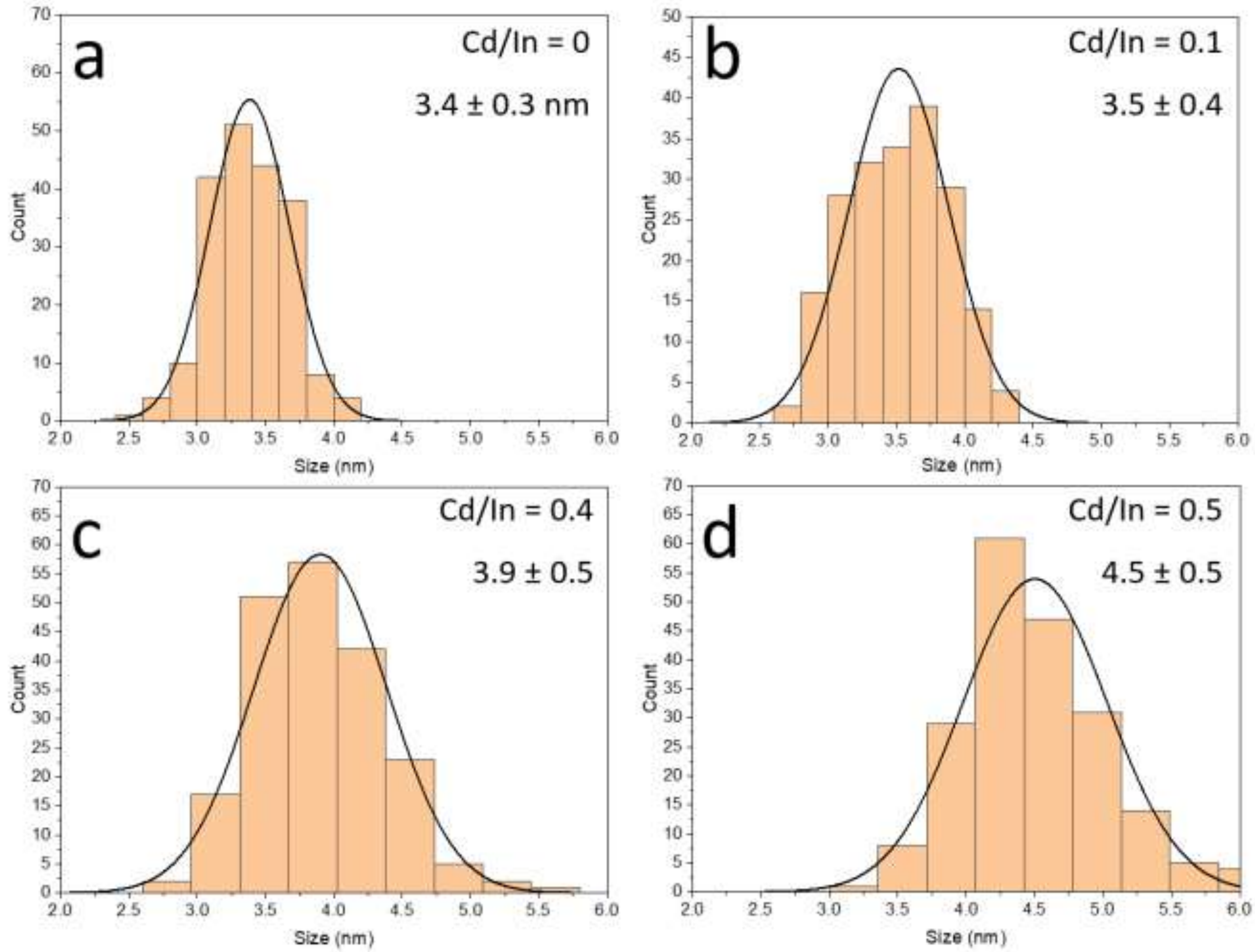

Figure S3. Size distribution histograms of (a) InAs and (b-d) In(Cd)As QDs made with different Cd/In precursor ratios.

S2. XPS analysis

Table S3. XPS quantitative data acquired for InAs, In(Cd)As and Cd-InAs QDs

Cd/In precursor ratio	In/As atomic ratio	Cd/In atomic ratio	Cd/As atomic ratio
0	1.03	0	0
0.4	0.95	0.34	0.32
0.5	0.90	0.37	0.34
$CdCl_2$-post treated	0.92	0.22	0.20

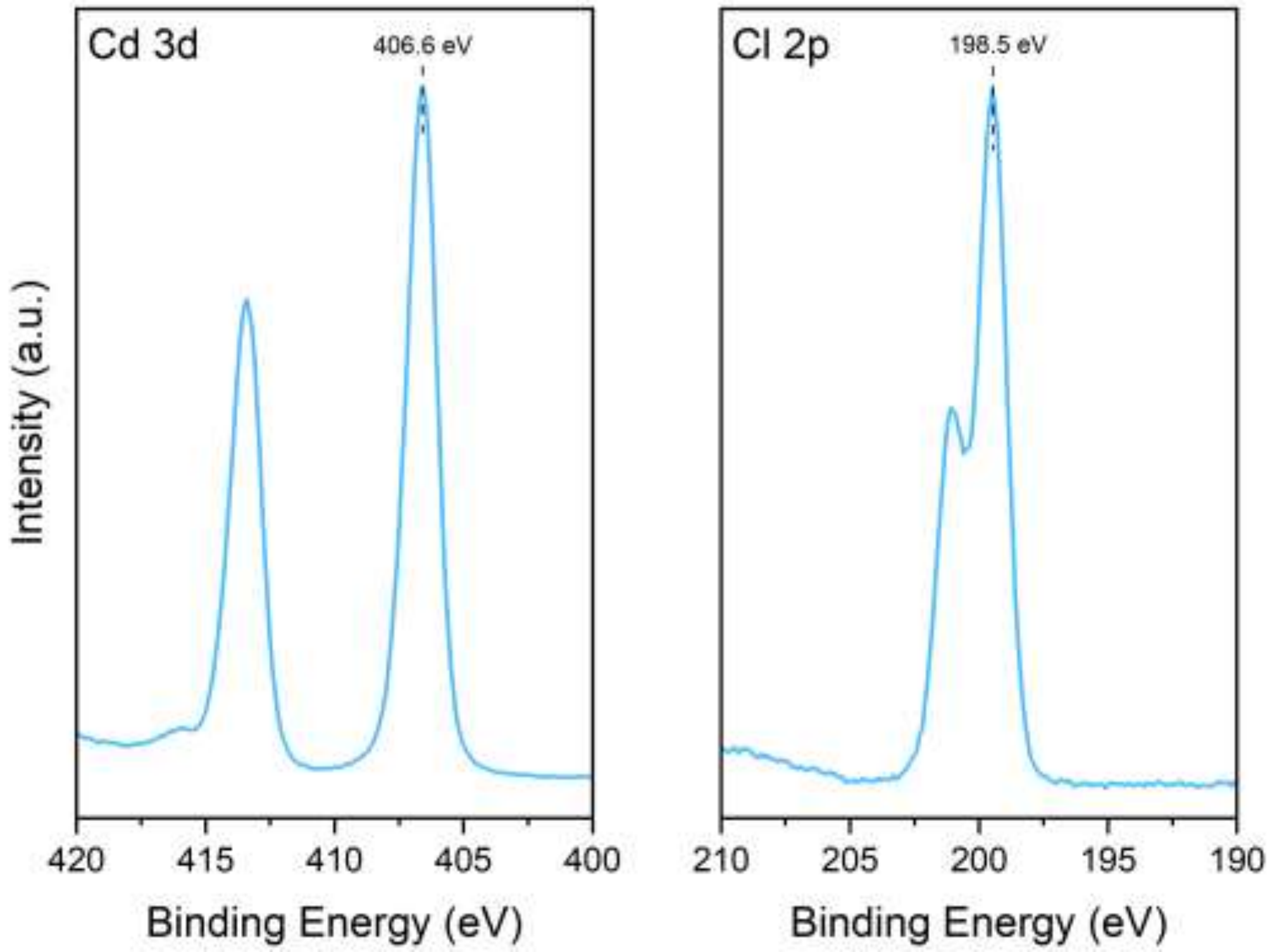

Figure S4. XPS analysis of $CdCl_2$: Cd 3d and Cl 2p spectra.

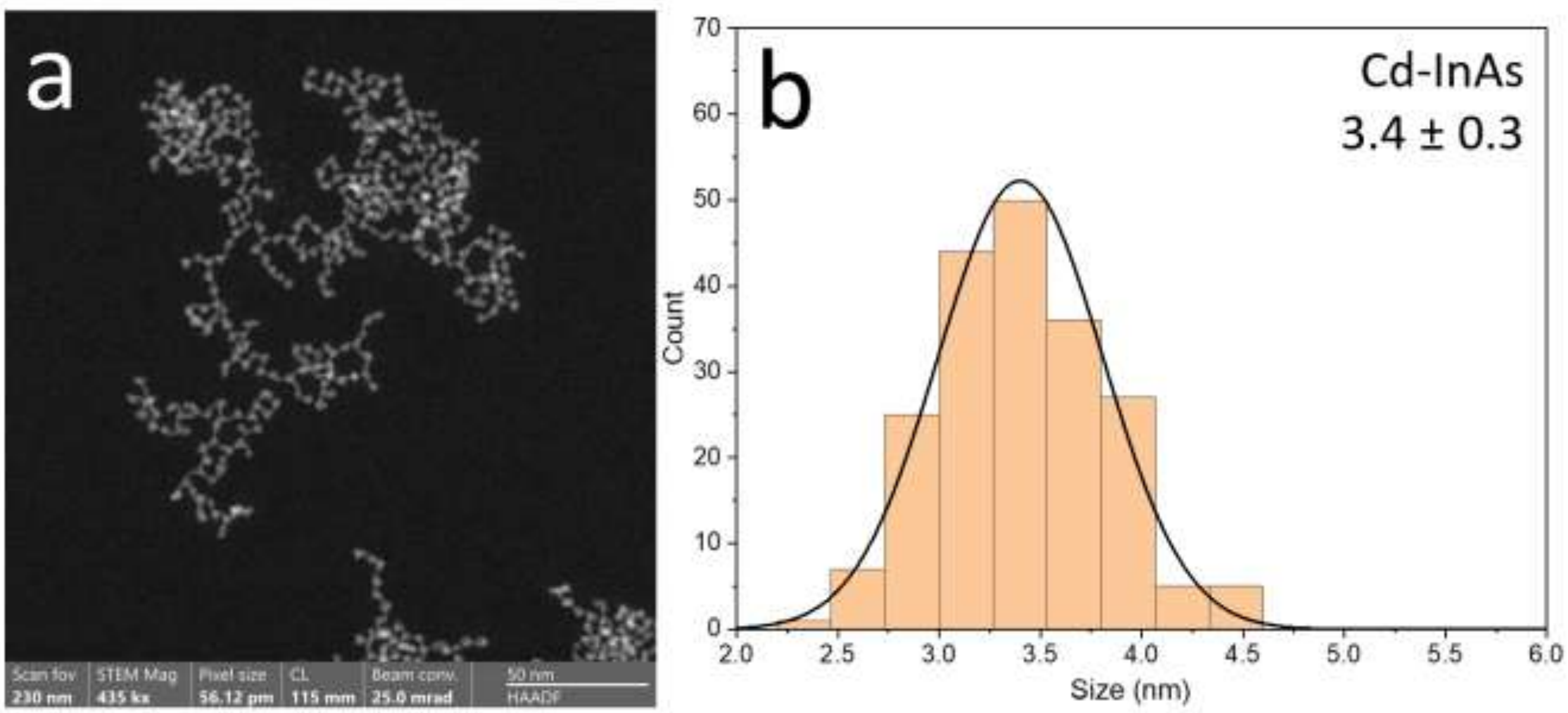

Figure S5. (a) HAADF-STEM image of Cd-InAs QDs and (b) the corresponding size distribution histogram.

S3. Optical properties

S3.1 PL decay and low fluence ΔA decay of InAs and Cd-InAs QDs.

Figure S6a shows the PL decay of InAs QDs. **Figure S6b** shows the decay of the bleach of InAs and Cd-InAs QDs at <N>=0.05. The bleach decay curves were fitted with a biexponential function. **Table S4** reports the fit parameters.

Table S4. **Fit parameters to the decay curves of Figure S6b.**

Sample	y_0	A_1	τ_1 (ps)	A_2	τ_2 (ps)
InAs	0.16	0.23±0.03	134±17	0.57±0.02	1105±93
Cd-InAs	0.12	0.32±0.02	106±9	0.54±0.02	983±75

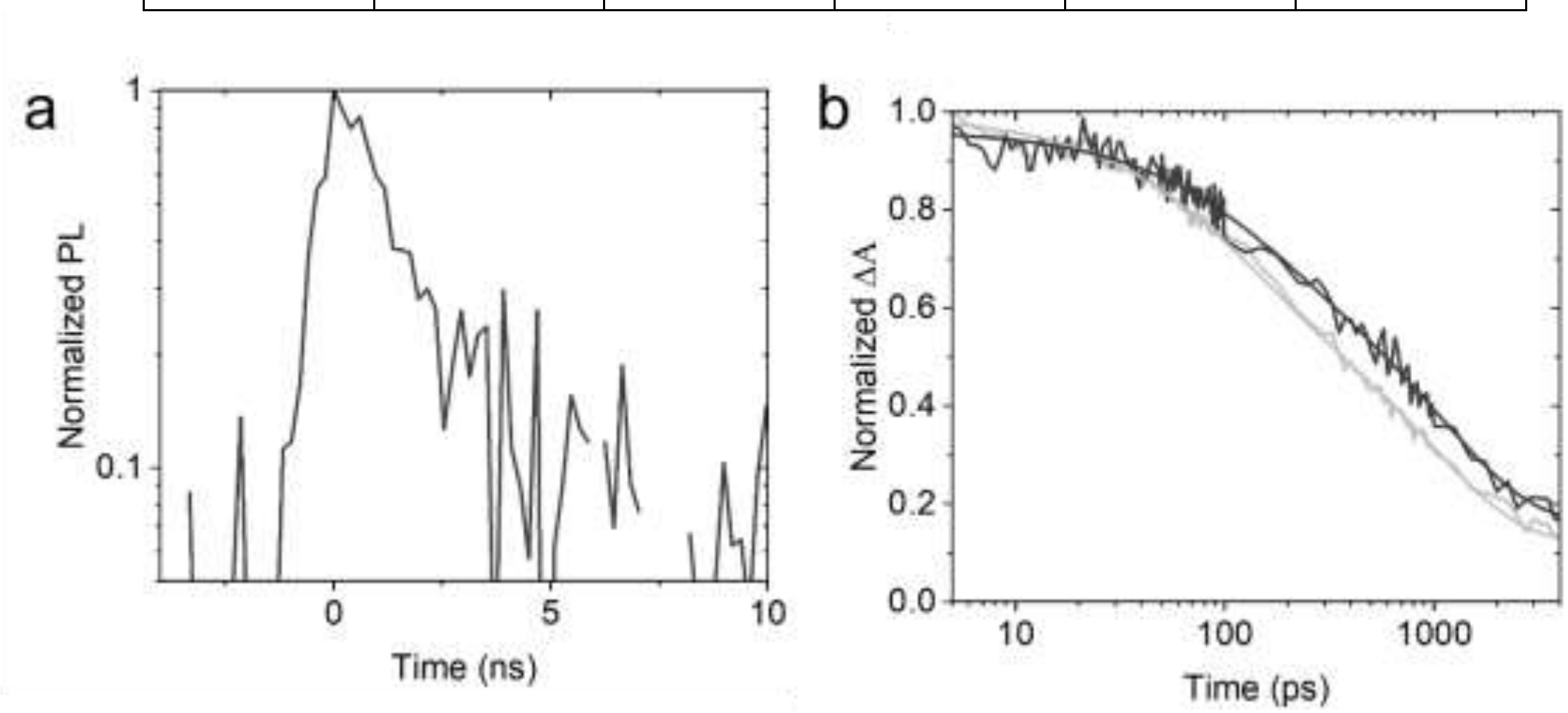

Figure S6. (a) PL decay of InAs QDs. (b) Fit of the bleach decay of InAs (black) and Cd-InAs (grey) QDs at <N>= 0.05.

S3.2 Biexciton lifetime of In(Cd)As QDs.

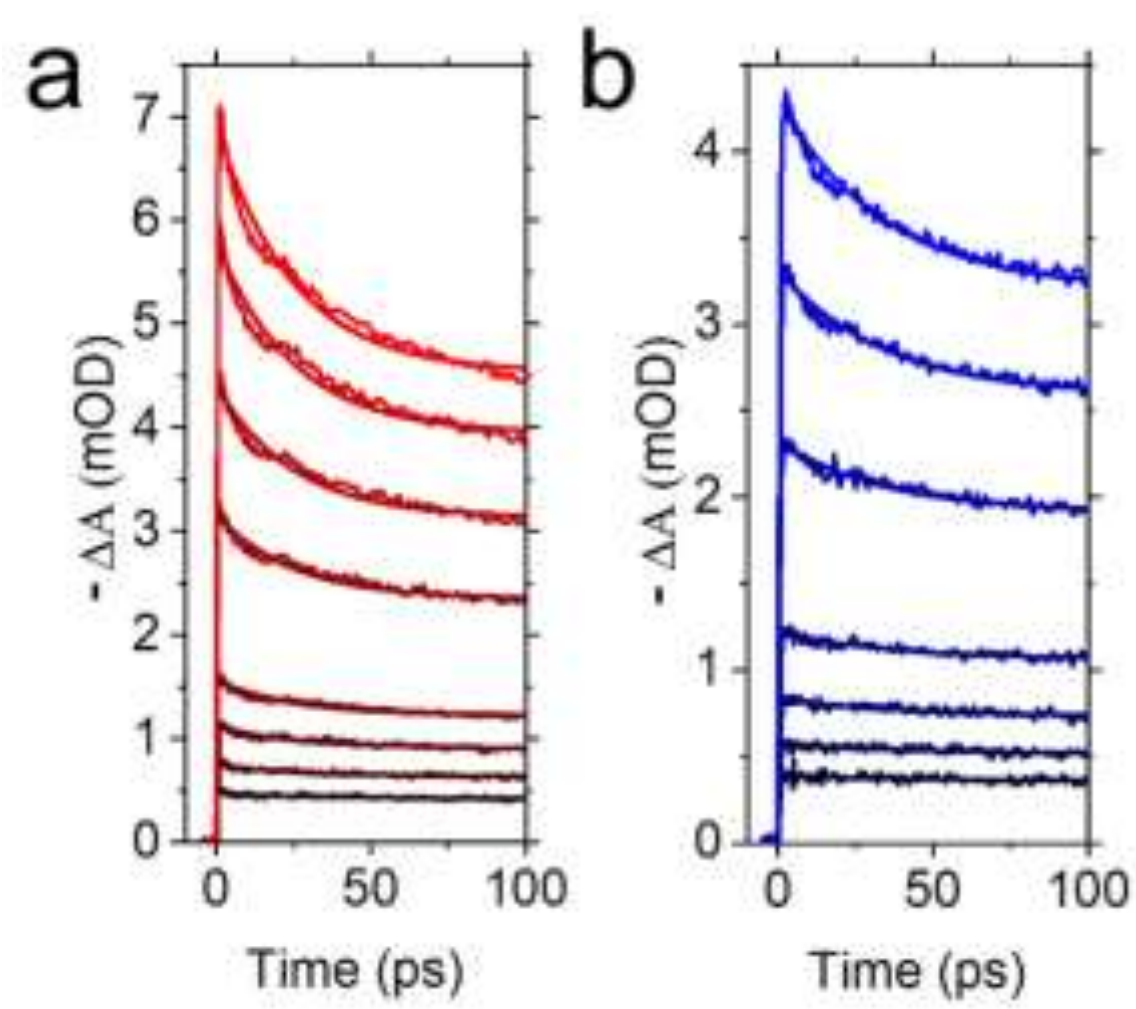

Figure S7. Global fit of the bleach decay of In(Cd)As QDs with (a) 0.1 (red) and (b) 0.4 (blue) Cd/In ratio obtained by increasing the fluence to generate <N> between 0.05 and 0.9.

S4. More computational details

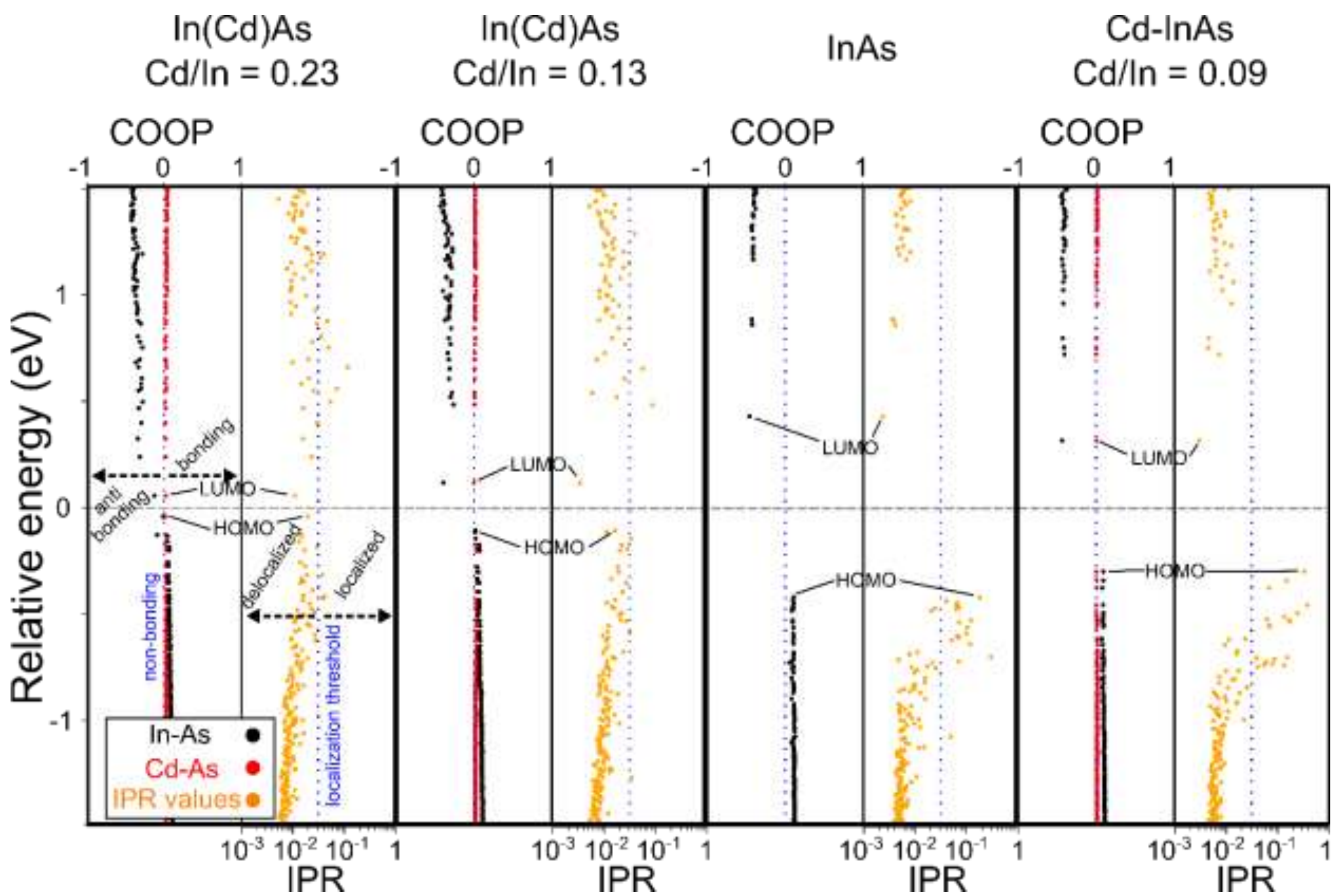

Figure S8. Crystal Orbital Overlap Population (COOP) plots evaluating In-As (black) and Cd-As (red) bond characters across a range from −1 (anti-bonding) to 1 (bonding); and Inverse Participation Ratio (IPR) plots mapping wavefunction localization from ~0 (delocalized) to 1 (highly localized) corresponding to In(Cd)As alloys (Cd/In = 0.23 and 0.13), InAs and the surface-substituted Cd-InAs, shown in **Figure 5b**. Vertical blue dotted lines denote the non-bonding baseline (0) for COOP plots, and the threshold separating delocalized and localized states for IPR plots. Note that the assignment of this IPR threshold is entirely subjective and selected visually according to the PDOS plots and wavefunction isosurfaces in **Figure 5b**, with a chosen value here of 3.1×10^{-2}. The horizontal grey dashed line in all plots marks the Fermi level, above (below) which MOs are unoccupied (occupied) and sets the energy origin.

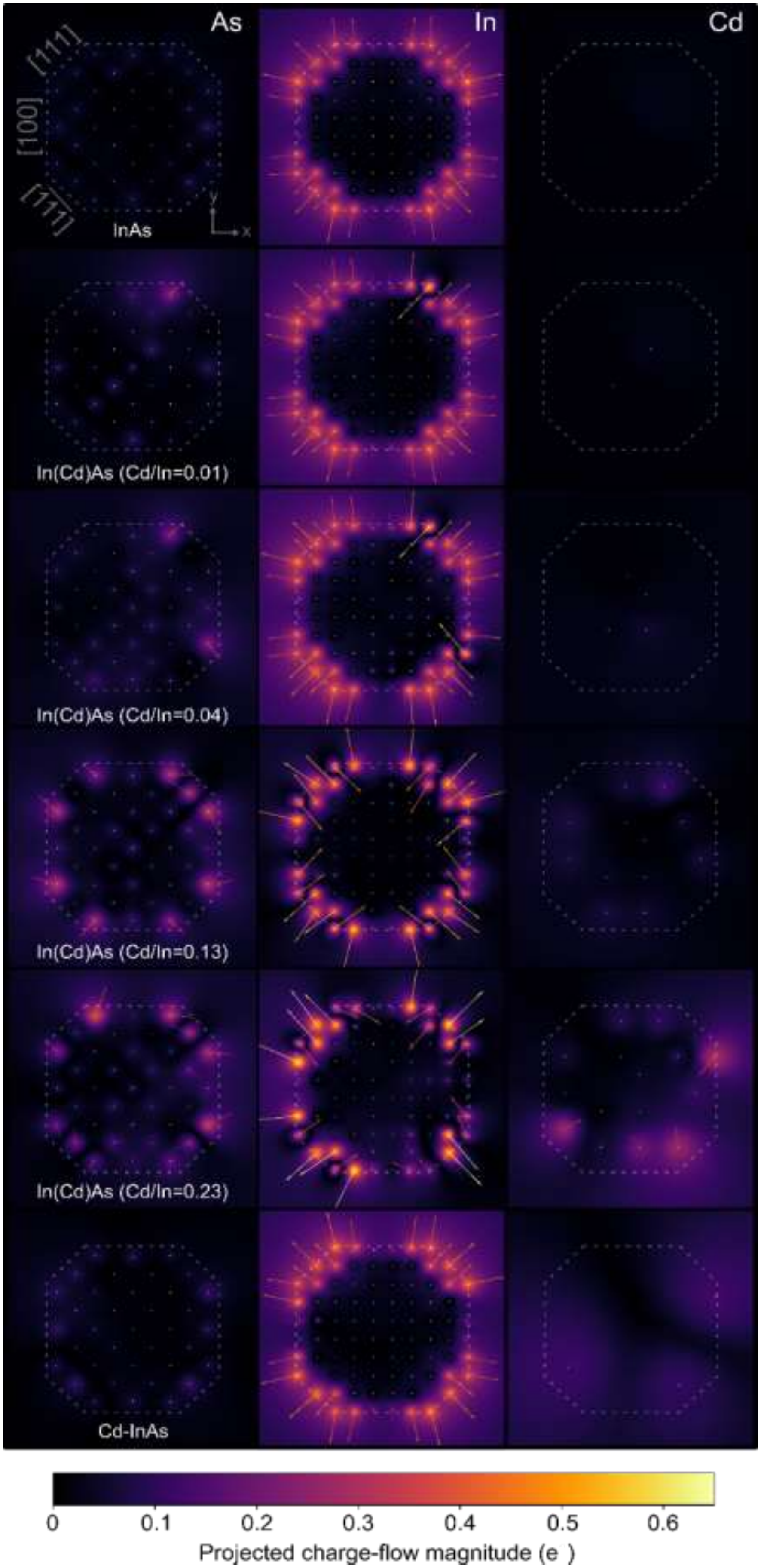

Figure S9. Charge-flow maps in the *xy*-plane. The eighteen panels show representative slices taken in the *xy*-plane and are chosen to include as many atoms as possible. The chosen cross-sectional slice thickness specifically encompasses three atomic planes – a central anionic layer flanked by upper and lower cationic layers – to fully capture the localized tetrahedral coordination environments, $AsIn_4$ and $AsIn_3Cd$. The columns correspond to the isolated sublattice contributions (arsenic – As, indium – In, and cadmium – Cd, left to right), and rows to the structural and doping progression (from top to bottom: pristine InAs baseline; core-alloyed In(Cd)As at Cd/In = 0.01, 0.04, 0.13, and 0.23 ratios; and post-treated Cd-InAs models). The white dots represent the atomic sites, while dashed grey octagonal outlines delimit the models' boundary. The superimposed vector arrows denote the per-atom charge-flow vectors: arrow direction indicates the net orientation of the induced local electronic flux, and arrow length reflects the vector magnitude. For the cation sublattices (In and Cd), inwardly directed arrows indicate charge transfer toward neighbouring As electron acceptors, whereas outwardly directed arrows reflect charge flow toward surface-passivating Cl ligands. The background maps show the smoothly interpolated magnitudes of such charge-flow vectors for better readability. Facet orientations ([100], [111], and [-1-1-1]) and coordinate axes are explicitly labelled in the top-left panel.

S4.1. Discussion on charge-flow analysis in Figure S9

The upward shift of the electronic structure originates from the reorganization of the internal electrostatic environment (**Figure S9**) and the resulting change in chemical bonding character (**Figure S8-COOP**). In the symmetric $AsIn_4$ atomic environments of the pristine InAs QD model, the electronic density is evenly distributed across the four equivalent In-As bonds. Considered as vectors, these charge flows compensate, yielding a stable, unpolarized central As atom (**Figure S9, InAs**). When Cd replaces In, forming the asymmetric $AsIn_3Cd$ environment, this electrostatic symmetry is broken. Because Cd possesses a smaller pool of valence electrons than In and the Cd-As bonds exhibit a quasi-non-bonding character, Cd acts as a spectator (**Figure S8-COOP**). This is visually corroborated by the Cd sublattices in **Figure S9** (**Cd column**), with Cd exhibiting relatively weak charge-flow magnitudes across all alloying ratios. Consequently, the central As atoms in the $AsIn_3Cd$ atomic environments receive less electron density from the Cd sites compared to the original In host cation in $AsIn_4$. To compensate for this localized charge deficiency, the central As atoms undergo a form of 'ghost undercoordination' and withdraw electron density more intensely from their three remaining In neighbors. This compensatory mechanism can be directly mapped in the In sublattices of **Figure S9**: as the alloying degree increases (from Cd/In = 0.01 to 0.23), the charge-flow vectors directed towards the As atomic centers (inwards) begin to elongate, while the As panels concurrently exhibit intense, localized charge accumulation. This asymmetric withdrawal severely weakens the primary In-As bonds, shifting their VB character from a bonding to a less bonding state. A less pronounced shift is observed in the CB In-As bond character, which largely preserves their antibonding nature (**Figure S8-COOP**). Experimentally, this localized bond weakening is consistent with the redshift of the InAs longitudinal optical (LO) phonon mode observed in the Raman spectra of the alloyed In(Cd)As QDs (**Figure 2b**). Unlike the internally alloyed regime, the Cd-InAs system introduces the substituted Cd atoms to the QD surface. Because this spatial Cd segregation preserves the internal $AsIn_4$ structural environments within the core, the internal electronic density remains evenly distributed as in the InAs case (**Figure S9**). Consequently, the charge-flow vectors across the core In-As bonds effectively compensate, entirely averting the 'ghost undercoordination' effect. Additionally, as evidenced by the preserved bonding character in the COOP profiles (**Figure S8-COOP)** and the same localization character in the IPR analysis (**Figure S8-IPR**), the primary internal In-As bonds in the Cd-InAs system largely maintain their pristine electrostatic and structural integrity. This is indeed supported by the experimental Raman spectra (**Figure 3c**), which show no phonon redshift for the surface-treated QDs.

S4.2. Thermodynamics of Cd Incorporation in InAs QDs and InAs bulk structures.

To understand if In-Cd-As alloys can form in the bulk and/or at the nanoscale, we evaluated the thermodynamics of Cd incorporation in both regimes. Due to the marked differences between finite nanocrystals and infinite periodic lattices, two different thermodynamic mechanisms were studied.

S4.2.1. QD Alloy Formation Thermodynamics (Exothermic Regime)

We assessed the thermodynamic feasibility of the formation of In(Cd)As alloyed QDs, which are observed via *direct synthesis* of InAs with $CdCl_2$ as an additive, and of Cd-InAs QDs, characterized by Cd atoms replacing In ones at the surface of the QDs, which form upon *post-synthesis surface treatment* of InAs QDs with $CdCl_2$. Because QDs are finite, non-periodic systems, their stoichiometry is fully defined, allowing us to evaluate thermodynamics using closed chemical equations without relying on chemical potential reservoirs required for bulk-system formation.

The baseline pristine InAs QD and the doped QDs (alloyed or surface-treated) are constructed from stoichiometric combinations of InAs monomers, $InCl_3$, $CdCl_2$, and methylamine (CH_3NH_2, abbreviated as MA) passivating ligands. Crucially, each Cd^{2+} for In^{3+} replacement is mediated by the removal of a surface Cl^- (computationally corresponding to the removal of an $InCl_3$ unit and the addition of a $CdCl_2$ unit, **Figure**

S10a), to keep the system charge balanced, and the addition of a neutral MA ligand, mimicking the experimental oleylamine, to passivate the newly exposed surface site (i.e. the one forming upon Cl^- removal). The formation of these systems can be expressed via the following general chemical equations:

$$(a-b)[InCl_3] + c[MA] + b[InAs] \rightarrow [In_aAs_b\text{-}\{3(a-b)[Cl];\, c[MA]\}](QD) \qquad \textit{InAs QD formation}$$

$$(a-b-d)[InCl_3] + (c+d)[MA] + d[CdCl_2] + b[InAs] \rightarrow [In_{(a-d)}Cd_dAs_b\text{-}\{(3(a-b)-d)[Cl];\, (c+d)[MA]\}](QD) \qquad \textit{Cd-incorporated InAs QD formation}$$

In these equations, square brackets […] denote specific chemical species or monomers, while (QD) indicates the assembled quantum dot. Within the QD, curly braces {…} specifically designate the surface-attached passivating ligands. The stoichiometric variables *a*, *b* and *c* represent the total number of In atoms, As atoms and MA ligands in the pristine QD, respectively, while *d* defines the number of incorporated Cd atoms. The full stoichiometric accounting of these precursor monomers across all models used in this work is detailed in Table S5, which explicitly demonstrates the mathematical conservation of [InAs] units.

Table S5. Elemental analysis and stoichiometric accounting of the chemical precursor monomers used to construct the pristine and Cd-incorporated InAs QDs.

		Elemental Analysis							Precursor Monomers			
**Model**	**Cd/In ratio**	**In**	**As**	**Cd**	**Cl**	**C**	**N**	**H**	**[InAs]**	**[InCl₃]**	**[CdCl₂]**	**[MA]**
InAs	0.00	336	273	0	189	15	15	75	273	63	0	15
In(Cd)As	0.01	332	273	4	185	19	19	95	273	59	4	19
	0.04	324	273	12	177	27	27	135	273	51	12	27
	0.13	297	273	39	150	54	54	270	273	24	39	54
	0.23	273	273	63	126	78	78	390	273	0	63	78
Cd-InAs	0.09	307	273	29	160	44	44	220	273	34	29	44

For example, for the pristine InAs QD ($a = 336$, $b = 273$, $c = 15$), this gives $(a - b) = 63$ units of $InCl_3$, $c = 15$ units of MA, and $b = 273$ units of InAs – exactly the precursor stoichiometry listed in **Table S5**, with a corresponding surface Cl content of $3(a - b) = 189$, matching the tabulated elemental analysis. Reading across the table, this surface composition changes gradually to 126 Cl^- and 78 MA ligands at Cd/In = 0.23, or to 160 Cl^- and 44 MA ligands in Cd-InAs, so that the total ligand count remains constant at 204 in all models.

To determine the relative thermodynamic cost of introducing Cd, we subtract the *InAs QD formation* chemical equation from the *Cd-incorporated InAs QD formation* chemical equation. Importantly, in our models, the substitution of In^{3+} with Cd^{2+} is strictly limited to the excess In pool (in $InCl_3$ form), while keeping constant the stoichiometric amount of [InAs] at all instances. Because the amount of [InAs] is identical in both formation equations, it cancels upon subtraction, leaving a net exchange of *d* units of $CdCl_2$ (plus *d* MA) for *d* units of $InCl_3$ – equivalent to assuming the excess $InCl_3$ is fully exchanged with $CdCl_2$.

The chemical exchange processes that model the alloying via *direct synthesis* and the *post-synthesis surface treatment* are described by the following balanced chemical equations, respectively:

$$InAs_{[pristine]} + d\,(CdCl_2 + MA) \rightarrow In(Cd)As_{[alloyed]} + d\,InCl_3 \qquad (\textit{direct synthesis}\text{ alloying})$$

$$InAs_{[pristine]} + d\,(CdCl_2 + MA) \rightarrow Cd\text{-}InAs_{[Cd@surface]} + d\,InCl_3 \qquad (\textit{post-synthesis surface treatment})$$

Hence, instead of using formation energy equations, we evaluated the substitution energy (ΔE_{subs}, Eq. (1)), defined as the total energy change associated with the stoichiometric exchange of native In^{3+} for Cd^{2+} in $InCl_3$ and $CdCl_2$ forms, respectively, for each of the two systems. Here, *d* denotes the total number of substituted cation sites within the ~3 nm tetrahedral InAs framework. The total energetic change (ΔE_{subs}) is calculated directly from the ground-state total energies derived from the DFT calculations:

$$\Delta E_{subs} = \{(E_{[alloyed\text{-}Cd@surface]} + dE_{[InCl_3]}) - (E_{[pristine]} + d(E_{[CdCl_2]} + E_{[MA]}))\} \qquad (1)$$

To facilitate a direct, concentration-independent comparison between systems with varying dopant densities, the final values reported in the main text are normalized per added $CdCl_2$ monomer:

$$(\Delta E_{subs})^{norm} = \Delta E_{subs} / d \qquad (2)$$

The calculated raw energy differences, ΔE_{subs} and their corresponding normalized values are summarized in **Table S6**, where the incorporation of Cd into and onto the QD is an exothermic process across all tested Cd/In ratios.

Table S6. Total number of substituted cation sites (*d*), total substitution energy (ΔE_{subs}), and normalized substitution energies per monomer, $(\Delta E_{subs})^{norm}$, corresponding to the different models used in this work. For the alloyed In(Cd)As models, the degree of substitution increases progressively from the most central subshell (Cd/In = 0.01) to the most external (Cd/In = 0.23).

Model	Cd/In ratio	*d* $CdCl_2$	ΔE_{subs} [kcal·mol^{-1}]	$(\Delta E_{subs})^{norm}$ [kcal·mol^{-1} · (d $CdCl_2$)$^{-1}$]
In(Cd)As	0.01	4	−71	−18
	0.04	12	−177	−15
	0.13	39	−314	−8
	0.23	63	−119	−2
Cd-InAs	0.09	29	−485	−17

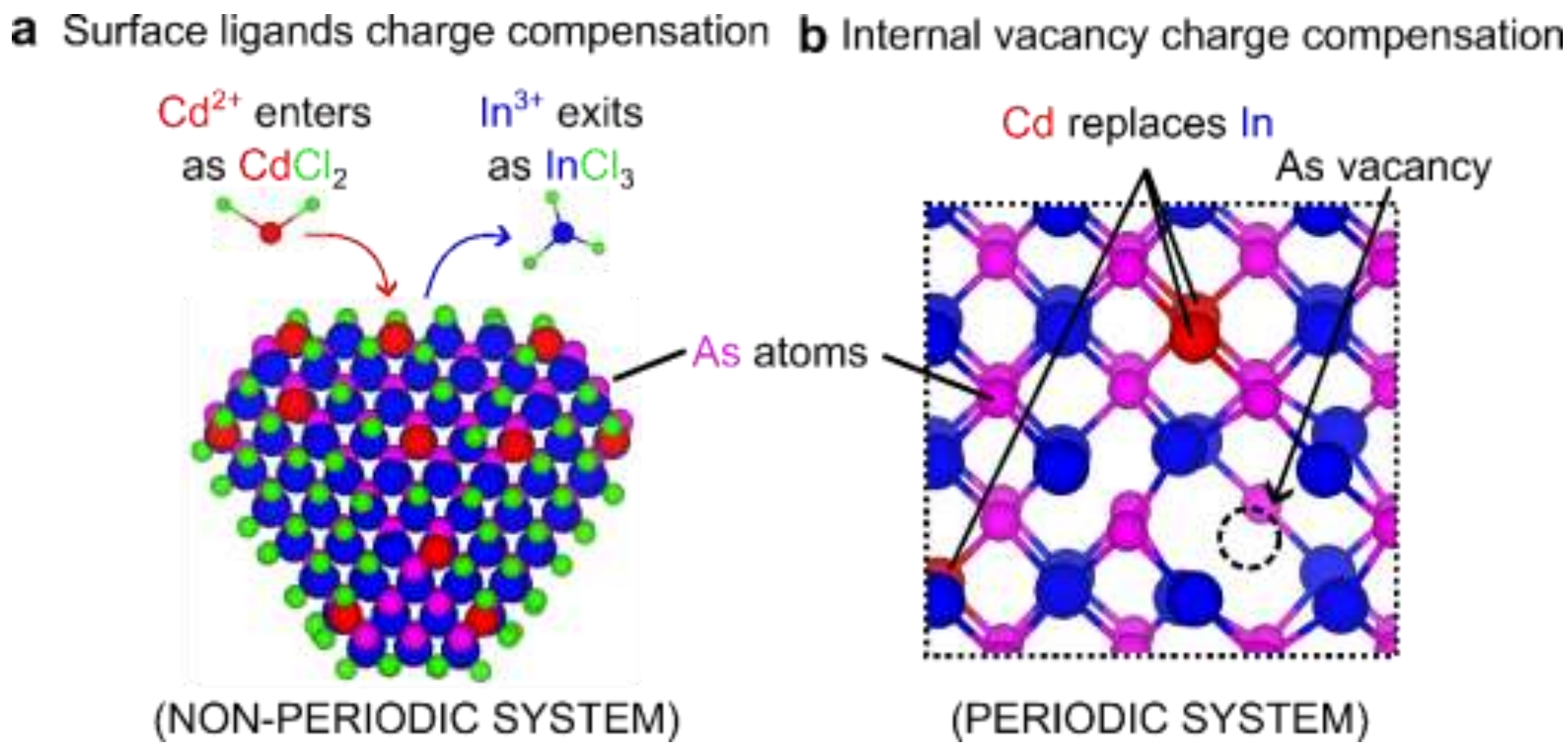

Figure S10. Schematic representation of the distinct charge compensation mechanisms upon Cd^{2+} incorporation into an InAs host: (a) QD: a confined and non-periodic system where the charge imbalance is resolved via surface ligand dynamics, illustrated by the generalized stoichiometric exchange of $CdCl_2$ and $InCl_3$ precursors. MA ligands have been omitted for visual clarity. (b) Bulk: a periodic system in which the absence of surface degrees of freedom leads to a need for internal structural reconstruction to charge-balance the system. This is achieved by the formation of one As vacancy for every three Cd^{2+} substitutions.

S4.2.2. Bulk Alloy Formation Limits (Endothermic Regime)

In an infinite periodic bulk lattice, the surface degrees of freedom required to mitigate the charge imbalance when replacing In^{3+} with Cd^{2+} sites are absent. In a QD, the relative −1 effective charge excess per Cd substitution can be easily resolved through the desorption of negatively charged ligands. Given the experimental use of metal chloride precursors ($CdCl_2$ and $InCl_3$) and neutral oleylamine, chloride ions (Cl^-) are the primary anionic species available in the environment to facilitate this compensating mechanism[2] (**Figure S10a**). In the bulk, however, this charge imbalance cannot be solved via ligand dynamics. Thus, the bulk lattice must instead undergo internal structural reconstruction to achieve charge neutrality; the substitution of three In atoms with three Cd atoms (creating a net −3 effective charge excess) necessitates the spontaneous generation of one As vacancy (yielding a +3 effective charge); see **Figure S10b**. To prove that this required internal reconstruction blocks the formation of an In(Cd)As alloy in the bulk, we evaluated the absolute formation energy (ΔE_f) of the fully compensated defect complex in **Figure S10b** using a chemical potential boundary formalism. Because the periodic calculations were evaluated strictly at the Γ-point, we replicated standard *k*-point sampling by expanding all primitive cells into 54-64 atom supercells, matching the dimensions of the 64-atom InAs host (cell side lengths of ~11-13 Å). This real-space expansion folds the Brillouin zone, making a Γ-point evaluation of a supercell mathematically equivalent to a *k*-point mesh of a primitive cell. With all reference supercells nearly sharing the same volume, their effective reciprocal-space sampling density may be considered identical, ensuring that finite-size integration errors largely cancel out when calculating ΔE_f.

Within this boundary formalism, ΔE_f is calculated by defining the energy cost of exchanging atoms with their respective environmental reservoirs. This is grounded in the host stability constraint ($\mu_{In} + \mu_{As} = E_{InAs}$), which defines the absolute formation energy as:

$$\Delta E_f = E_{vacs} - E_{pristine} + 3\mu_{In} + \mu_{As} - 3\mu_{Cd} \equiv E_{vacs} - E_{pristine} + 2\mu_{In} + E_{InAs} - 3\mu_{Cd} \quad (3)$$

Here, E_{vacs} and $E_{pristine}$ are the absolute DFT ground-state total energies of the defective In(Cd)As (63-atom) and pristine InAs (64-atom) supercells, respectively (**Figure S11)**. The variable μ_x denotes the chemical potential of species x, reflecting changes in environmental conditions during synthesis. The term E_{InAs} represents the calculated energy of a single In-As stoichiometric pair within the bulk lattice, defined as $E_{pristine}$ / 32.

To provide the absolute most favourable theoretical conditions for alloy formation, a Cd-saturated environment was assumed ($\mu_{Cd} = E_{Cd}$, **Figure S11**), and ΔE_f was evaluated at the extremes of the host chemical potential window:

1. As-rich limit (theoretical best-case scenario):

Here, three In atoms must be extracted for each As atom removed. In an As-rich environment, the energetic penalty for In extraction is minimized, as the extracted In can eventually react with the excess As atoms. Also, μ_{As} is constrained by the energy per atom of the bulk grey As phase ($\mu_{As} = E_{As}$, **Figure S11**), which forces $\mu_{In} = E_{InAs} - E_{As}$,

$$\Delta E_{f,\ \text{As-rich}} = E_{vacs} - E_{pristine} + 3E_{InAs} - 2E_{As} - 3E_{Cd} \quad (4)$$

2. In-rich limit (theoretical worst-case scenario):

Conversely, in an environment saturated with In atoms, the thermodynamic resistance for extracting In atoms from the lattice is maximized, as the In-rich environment is expected to resist the incorporation of more In, resulting in the highest energy penalty. Here, μ_{In} is constrained by the energy per atom of the bulk metallic In phase ($\mu_{In} = E_{In}$, **Figure S11**), which forces $\mu_{As} = E_{InAs} - E_{In}$,

$$\Delta E_{f,\ \text{In-rich}} = E_{vacs} - E_{pristine} + 2E_{In} + E_{InAs} - 3E_{Cd} \quad (5)$$

The calculated ΔE_f values at both boundaries are indeed endothermic (~42 kcal/mol and ~74 kcal/mol, respectively). These energy penalties demonstrate that the internal structural reconstruction required to charge-balance the bulk In(Cd)As by means of As vacancies completely suppresses the formation of a bulk solid solution.

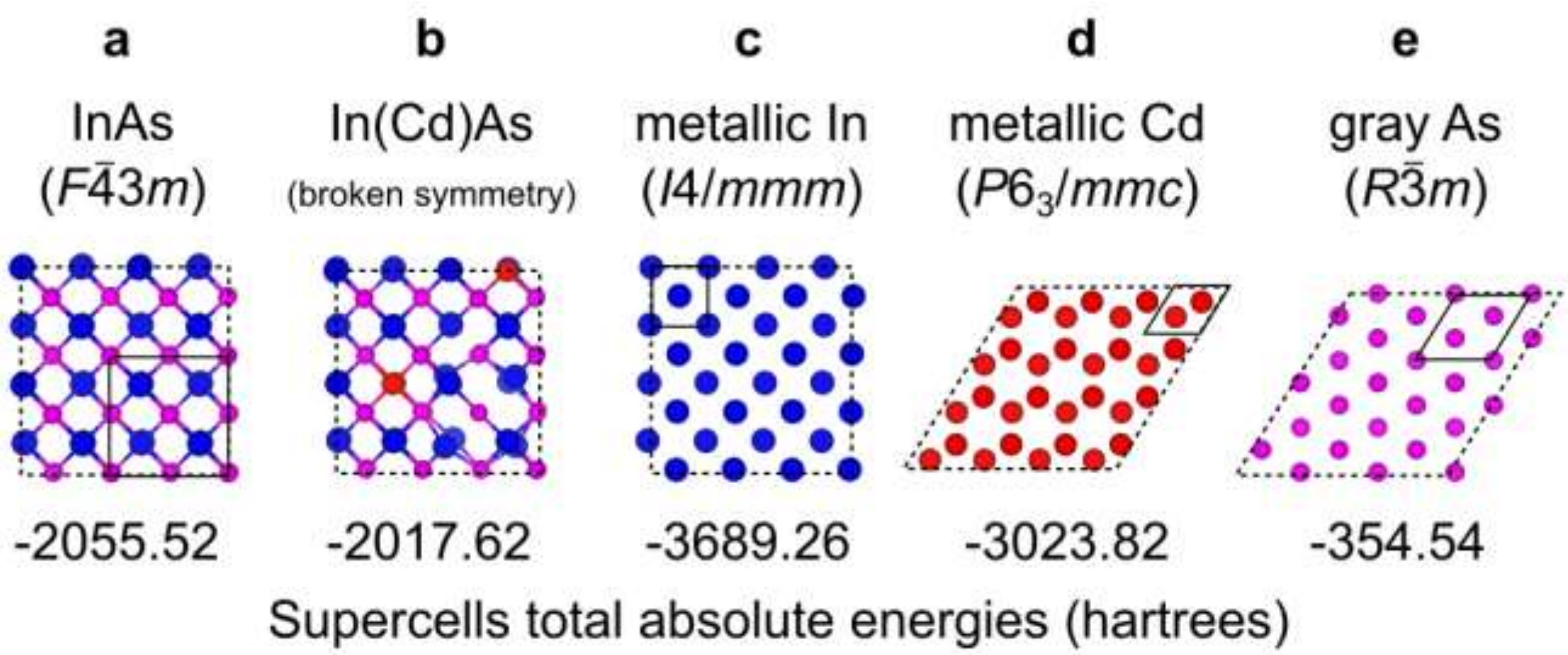

Figure S11. Schematic projections of (a) the pristine 64-atom InAs ($F\bar{4}3m$) host supercell and (b) the alloyed 63-atom In(Cd)As supercell. Standard elemental reference phases are shown for (c) the metallic 64-atom In ($I4/mmm$), (d) metallic 64-atom Cd ($P6_3/mmc$), and (e) 54-atom gray As ($R\bar{3}m$). Solid

black outlines denote the primitive unit cells for the standard phases, while dashed lines delineate the boundaries of the supercells. The listed values represent the absolute ground-state total energies (in hartrees) of these respective supercells, providing the raw values for $E_{pristine}$, E_{vacs}, and the elemental references (E_{In}, E_{Cd}, E_{As}). To evaluate ΔE_f in Equations (4) and (5), the elemental references must be normalized to their per-atom energies (μ_x): $E_{In} = -57.645$ hartrees (from 64 atoms), $E_{Cd} = -47.247$ hartrees (from 64 atoms), and $E_{As} = -6.566$ hartrees (from 54 atoms). Initial crystallographic coordinates for generating the supercells were obtained from the Materials Project database.[3,4]

The stark contrast between the exothermic nanoscale substitution (**Table S6**) and the highly endothermic bulk limiting cases supports the conclusion that In(Cd)As and Cd-InAs are stabilized by finite-size effects, rendering their existence in bulk periodic crystals thermodynamically highly unfavorable. This is consistent with the experimental absence of bulk In–Cd–As alloys in crystallographic databases.

S4.3. Local structural analysis

Pair-distance distributions were computed from the optimized geometries. All In–As and Cd–As interatomic distances up to 7.25 Å were collected into a histogram and smoothed with a Gaussian kernel (σ = 0.06 Å). Each curve was divided by the number of pairs it contains, so that the area under every curve is the same and models of different compositions can be compared directly. First-shell means and standard deviations were obtained over distances below 3.0 Å, which isolates the nearest-neighbor peak from the second shell at ~5.0 Å.

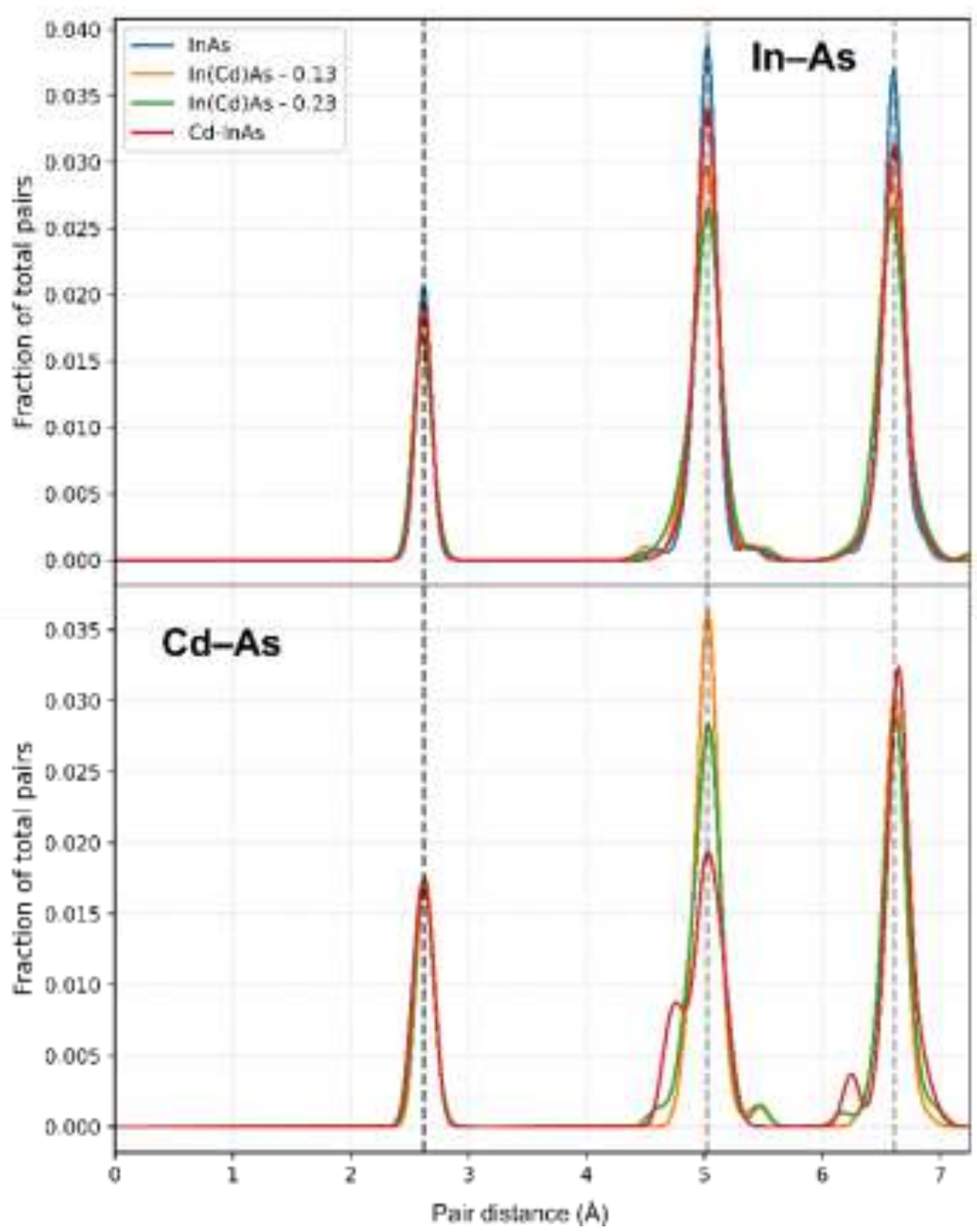

Figure S12. In-As (top) and Cd-As (bottom) pair-distance distributions for the optimized InAs (blue lines), In(Cd)As (Cd/In = 0.13 and 0.23 – orange and green lines, respectively) and Cd-InAs models (red lines). Dashed vertical lines mark the first three coordination shells of pristine InAs and are common to both panels.

The first-shell In-As distance is essentially unchanged from the pristine value (2.617 Å) in every model, while the Cd-As distance falls in the same range. The second and third shells are likewise unshifted, indicating that Cd substitution is isostructural. The distributions broaden slightly upon core alloying, reflecting some local bond disorder rather than a net lattice deformation, while the surface-substituted Cd-InAs model remains as narrow as the pristine InAs one. The additional features between the main peaks arise from atoms at the surface that lack a complete coordination shell; they are visible in both panels but are more pronounced in the Cd-As distributions, where the surface contribution represents a larger fraction of the total pairs.

S4.4. Absolute alignment of the frontier orbital energies

Because the zero of the electrostatic potential is set by the electrostatic treatment of each calculation rather than by a physical asymptote, eigenvalues from separate calculations are not directly comparable. We therefore introduced a non-interacting H_2 molecule in the vacuum region of each model and used its occupied σ orbital as a common reference. H_2 was chosen because its σ level lies well below the valence manifold and its σ* well above the conduction manifold, so that it cannot contribute states near the gap.
An identical H_2 molecule, with the same internal geometry, was placed at the same distance and orientation relative to the center of every model, approximately 9 Å beyond the outermost atom and no closer than 12 Å to any QD atom. Since the spectator and the QD belong to the same calculation, the arbitrary offset of the electrostatic potential cancels when each eigenvalue is measured relative to the σ level, placing all four models on a common absolute scale. Frontier orbitals were identified as the highest occupied and lowest unoccupied molecular orbitals (HOMO and LUMO), since the electron counts differ between models.

Table S7. Energy shifts of the HOMO and LUMO of each model relative to the corresponding level in InAs, in eV.

Model	ΔE_{HOMO}	ΔE_{LUMO}
**InAs**	0.00	0.00
**In(Cd)As (Cd/In = 0.13)**	+1.98	+1.34
**In(Cd)As (Cd/In = 0.23)**	+2.46	+1.67
**Cd-InAs (Cd/In = 0.09)**	+0.59	+0.38

These results support the bonding/anti-bonding picture deduced from the COOP analysis. Cd incorporation destabilizes both band edges, with the valence edge being considerably more perturbed than the conduction edge, so that the gap contraction is asymmetric and becomes increasingly so with Cd content. In the surface-substituted Cd-InAs model, both shifts are much smaller.

S4.5. Z-type $CdCl_2$ passivation of the Cd-InAs model

To assess the role of Z-type $CdCl_2$ passivation, an anion-rich (-1-1-1) facet of a Cd-InAs model with Cd/In = 0.20 was passivated by adsorbing $CdCl_2$ on its undercoordinated As atoms, and compared with the same facet left unpassivated. Both models are charge-balanced.

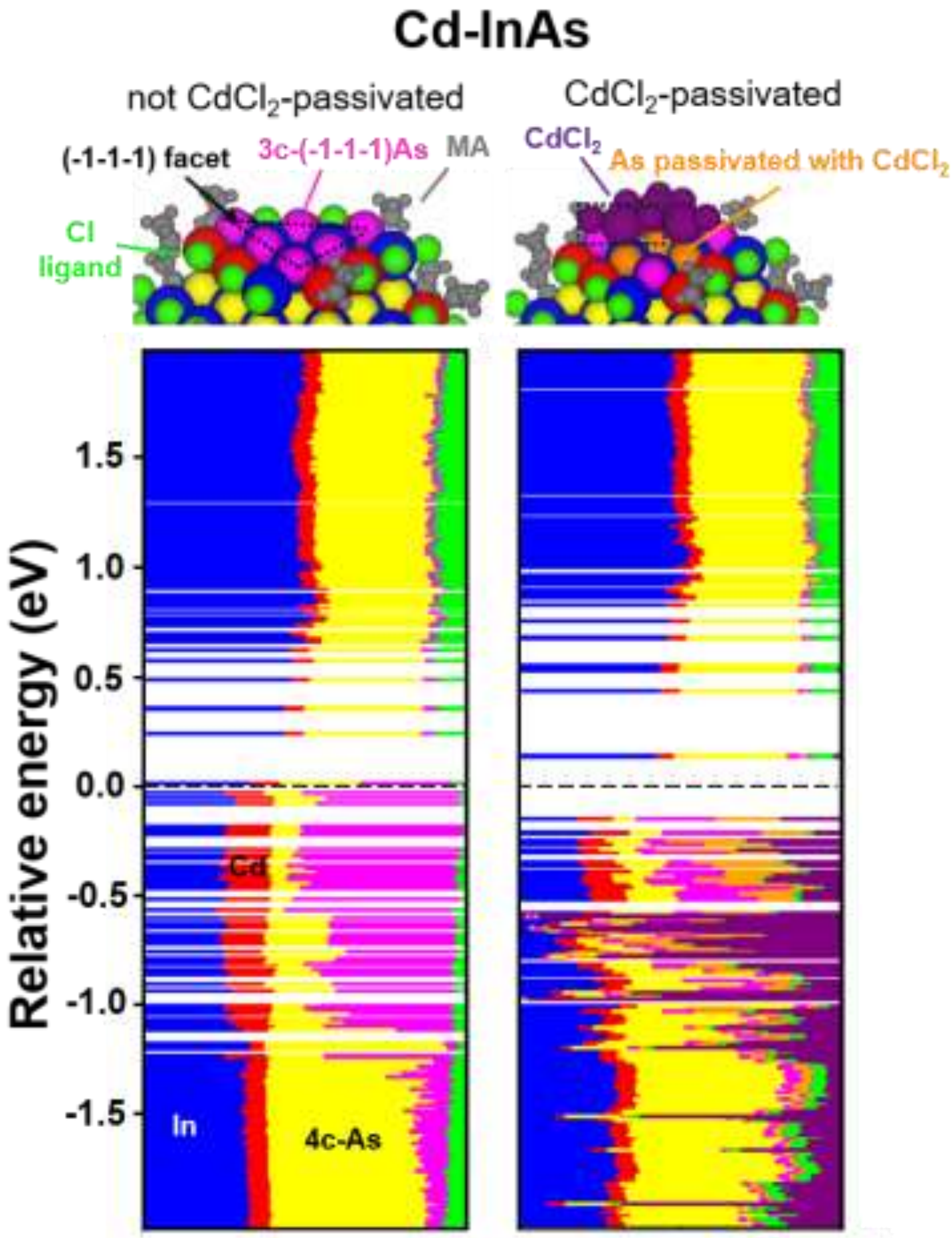

Figure S13. Structural models of the (-1-1-1) facet (top) and projected densities of states (bottom) of the Cd-InAs model (Cd/In = 0.20), unpassivated and with $CdCl_2$ adsorbed as a Z-type ligand on the undercoordinated As atoms. The As atoms coordinated by $CdCl_2$ retain frontier character immediately below the gap edge, essentially where the uncoordinated 3c-As states lie in the unpassivated model, while the $CdCl_2$ contribution itself appears deeper in the valence manifold. Energies are referenced to the midgap of each model, used here as a common plotting origin.

REFERENCES

(1) Panda, S.; Zhu, D.; Goldoni, L.; Asaithambi, A.; Brescia, R.; Saleh, G.; De Trizio, L.; Manna, L. Overcoming the Short-Wave Infrared Barrier in the Photoluminescence of Amino-As-Based InAs Quantum Dots. *Adv. Opt. Mater.* **2025**, *13*, e01512.

(2) Leemans, J.; Dümbgen, K. C.; Minjauw, M. M.; Zhao, Q.; Vantomme, A.; Infante, I.; Detavernier, C.; Hens, Z. Acid–Base Mediated Ligand Exchange on Near-Infrared Absorbing, Indium-Based III–V Colloidal Quantum Dots. *J. Am. Chem. Soc.* **2021**, *143*, 4290–4301.

(3) Jain, A.; Ong, S. P.; Hautier, G.; Chen, W.; Richards, W. D.; Dacek, S.; Cholia, S.; Gunter, D.; Skinner, D.; Ceder, G.; Persson, K. A. Commentary: The Materials Project: A Materials Genome Approach to Accelerating Materials Innovation. *APL Mater.* **2013**, *1*, 011002.

(4) Horton, M. K.; Huck, P.; Yang, R. X.; Munro, J. M.; Dwaraknath, S.; Ganose, A. M.; Kingsbury, R. S.; Wen, M.; Shen, J. X.; Mathis, T. S.; Kaplan, A. D.; Berket, K.; Riebesell, J.; George, J.; Rosen, A. S.; Spotte-Smith, E. W. C.; McDermott, M. J.; Cohen, O. A.; Dunn, A.; Kuner, M. C.; Rignanese, G.-M.; Petretto, G.; Waroquiers, D.; Griffin, S. M.; Neaton, J. B.; Chrzan, D. C.; Asta, M.; Hautier, G.; Cholia, S.; Ceder, G.; Ong, S. P.; Jain, A.; Persson, K. A. Accelerated Data-Driven Materials Science with the Materials Project. *Nat. Mater.* **2025**, *24* (10), 1522–1532.
